# Impact of COVID-19 school learning model on mental health, suicidal thoughts and behaviors, substance use, and violence related behaviors and experiences among U.S. high school students

**DOI:** 10.1371/journal.pmen.0000409

**Published:** 2024-09-17

**Authors:** David A. Katz, Sanjana Pampati, Sarah DeGue, Jean Y. Ko, Dawn Pepin, Casey E. Copen, Ari Fodeman, Deven T. Hamilton

**Affiliations:** 1 Department of Global Health, University of Washington, Seattle, Washington, United States of America,; 2 Division of Adolescent and School Health, National Center for Chronic Disease Prevention and Health Promotion, Centers for Disease Control and Prevention, Atlanta, Georgia, United States of America,; 3 Division of Violence Prevention, National Center for Injury Prevention and Control, Centers for Disease Control and Prevention, Atlanta, Georgia, United States of America,; 4 Division of Overdose Prevention, National Center for Injury Prevention and Control, Centers for Disease Control and Prevention, Atlanta, Georgia, United States of America,; 5 Program and Performance Improvement Office, National Center for HIV, Viral Hepatitis, STD, and Tuberculosis Prevention, Centers for Disease Control and Prevention, Atlanta, Georgia, United States of America,; 6 Division of STD Prevention, National Center for HIV, Viral Hepatitis, STD, and Tuberculosis Prevention, Centers for Disease Control and Prevention, Atlanta, Georgia, United States of America,; 7 Center for Studies in Demography and Ecology, University of Washington, Seattle, Washington, United States of America

## Abstract

The COVID-19 pandemic and associated prevention measures reshaped students’ lives. We estimated the effects of virtual learning during the pandemic on adolescents’ mental health, suicidal thoughts and behaviors, substance use, and violence-related experiences and behaviors. Using Youth Risk Behavior Survey data from 25 local jurisdictions, we compared overall and relative changes from 2019 (n = 49,740) to 2021 (n = 42,995) between jurisdictions where high school students predominantly attended school virtually vs. in-person during the 2020–2021 school year with Rao-Scott chi-square tests and difference-in-differences logistic regression. P-values<0.050 were considered significant. From 2019 to 2021, reported persistent feelings of sadness/hopelessness (2019 = 36.3%, 2021 = 41.3%), seriously considering suicide (17.0–18.7%), making suicide plan(s) (14.2–16.9%), and experiencing sexual dating violence in past 12 months (6.7–7.8%) increased significantly, while alcohol use (22.5–17.1%), binge drinking (8.5–6.2%), and marijuana use (20.2–14.3%) in past 30 days, ever prescription opioid misuse (16.0–13.2%), and gun-carrying (5.5–4.5%), physical fighting (22.3–17.3%), and any sexual violence (11.3–10.4%) in past 12 months decreased significantly. Students in virtual learning jurisdictions reported relative decreases in injury during suicide attempt(s) (OR=0.68, 95%CI = 0.52–0.88), experiencing sexual violence (OR=0.78, 95%CI = 0.67–0.91), using electronic vapor products (OR=0.81, 95%CI = 0.70–0.95), using alcohol (OR=0.84, 95%CI = 0.74–0.95), and misusing prescription opioids (OR=0.82, 95%CI = 0.72–0.94). Learning model was not associated with relative changes in persistent feelings of sadness/hopelessness, seriously considering suicide, making suicide plan(s), attempting suicide, binge drinking, marijuana use, gun-carrying, physical fighting, experiencing sexual dating violence, or being electronically bullied. Additional efforts to support schools in providing safe, supportive environments are needed, particularly where schools returned in-person in fall 2020.

## Introduction

The COVID-19 pandemic and associated prevention measures, including widespread closures of school buildings, substantially disrupted the lives and routines of adolescents. During the 2019–2020 school year, nearly all public school districts in the U.S. were closed for in-person instruction for an extended period of time [1]. Schools not only provide educational services but also have the potential to provide a safe and supportive environment; opportunities for the development of social and emotional learning skills; essential health and social services, including mental and behavioral health services; dedicated space and time for physical activity; and multiple meals a day [2]. After several professional organizations recommended returning to in-person school during the 2020–2021 school year and the Centers for Disease Control and Prevention (CDC) provided guidance for consideration for a safe return [3], school modality policies across the country became more varied, with a combination of in-person, remote, and hybrid learning options offered [4].

Evidence on the impact of prolonged school closures on adolescent health and behavioral outcomes, including mental health, substance use, and violence experiences, has been mixed. A systematic review found that school closures, as part of a broader range of COVID-19 prevention measures, were associated with adverse mental health symptoms (e.g., distress and anxiety) among children and adolescents [5,6]. In addition, some studies suggest that transitions to virtual learning were associated with reductions in specific forms of bullying [7,8]; however, experiences of cyberbullying may have increased as youth spent more time online [9]. A survey of student experiences during the 2020–2021 school year found that a higher percentage of students with in-person versus virtual learning had started or increased use of ≥1 substance to cope with stress or emotions [10]. Another study observed increases in emergency department visits for drug overdoses among adolescents during fall 2022 versus fall 2019, though data regarding school learning modality were not available [11].

Although these studies provide an important foundation, many are subject to important limitations, including using convenience-based sampling or non-experimental designs, ad-hoc data collection relying on reflections by adolescents or proxies on changes in outcomes, and not accounting for pre-existing secular trends. To address these limitations and examine longer-term impacts on adolescent health, we used a difference-in-differences (DID), quasi-experimental approach to estimate the impact of jurisdiction-level virtual (versus in-person) learning during the 2020–2021 school year on population-level mental health, suicidal thoughts and behaviors, substance use, and violence related behaviors and experiences among U.S. high school students using multiple years of data from the biennial population-based, representative Youth Risk Behavior Survey (YRBS). Understanding how COVID-19-related school closures and virtual learning may have impacted longer-term adolescent health can inform responses and recovery efforts for future public health emergencies involving schools and provide insight into the potential impacts of virtual vs. in-person learning models more generally.

## Methods

### Ethics statement

The University of Washington Human Subjects Division determined that these secondary analyses of de-identified or aggregated data did not meet the definition of human subjects research (STUDY00018010). Authors did not have access to information that could identify participants.

### Data source

Twenty-eight local jurisdictions participated in the YRBS–a biennial self-administered survey conducted in-person in schools by departments of health and education at the state, territorial, or local level–in both 2019 and 2021 [12]. YRBS collects self-reported health-related behaviors and experiences among representative samples of 9^th^-12^th^ grade students. Random sampling occurs in two stages. First, schools in the jurisdiction are selected with probability proportional to 9^th^–12^th^ grade enrollment. Second, classes are sampled from either a required subject or a required period. Individual schools and students can choose not to participate. Full details regarding YRBS methodology is available elsewhere [12], and the full questionnaires are available online at https://www.cdc.gov/yrbs/questionnaires/index.html. Data from 25 jurisdictions were available in the 2021 public-use dataset (accessed 7 November 2023) [13]; two additional jurisdictions provided data on request [Nashville, TN (accessed 15 November 2023); Boston, MA (accessed 23 May 2023)].

### Learning model ascertainment

To determine the predominant learning model (virtual versus in-person) for the 2020–2021 school year for high schools in local YRBS jurisdictions, we used data from the COVID-19 School Data Hub (CSDH; https://www.covidschooldatahub.com/). CSDH compiled information from state education agencies on learning models (virtual, hybrid, in-person) at the district- or school-level on a periodic basis. We prioritized district-level data [14] when available (13 of 28 districts), followed by school-level data [15] for all schools including 9^th^-12^th^-grade students in the relevant school district(s) (15 districts). When unable to determine a jurisdiction’s learning model using CSDH data, we reviewed government websites for relevant school, executive, or administrative policies and confirmed with additional sourced documentation from district websites or local news, similar to previous analyses [16,17]. When a jurisdiction included multiple school districts or schools within a jurisdiction reported different learning models, we attempted to summarize learning models across districts/schools for the YRBS jurisdiction.

Most jurisdictions categorized as “virtual” reported virtual learning from at or near the beginning of the 2020–2021 school year through at least February 2021. Some virtual jurisdictions moved to hybrid or in-person learning towards the end of the school year. Most jurisdictions categorized as “in-person” reported in-person learning for the entire school year. Two jurisdictions (Genesee Consortium and Eaton Consortium, MI) included several school districts with conflicting models during most of the year and, therefore, could not be categorized as predominantly virtual or in-person and were excluded from analyses. Details regarding learning model categorization are in S1 Table.

### Outcomes

Self-reported indicators of poor mental health and suicide were measured in the past 12 months and included: feeling so sad or hopeless almost every day for two weeks or more in a row that they stopped doing some usual activities (“persistent feelings of sadness or hopelessness”); seriously considering attempting suicide; making a suicide plan; attempting suicide; and having a suicide attempt result in an injury, poisoning, or overdose that had to be treated by a doctor or nurse (“injured in a suicide attempt”). Substance use outcomes included: electronic vapor product use; any alcohol use; binge drinking (having ≥4 drinks of alcohol in a row for females and ≥5 drinks of alcohol in a row for males); marijuana use in the past 30 days; and misusing prescription opioids in their lifetime. Violence related behaviors and experiences were measured in the past 12 months and included: carrying a gun (excluding for hunting or sport); having been in a physical fight; being forced to do sexual things they did not want to do (“experiencing any sexual violence”); experiencing sexual violence from someone they were dating (“sexual dating violence”); and being electronically bullied (e.g., via texting or social media). For all analyses, outcomes were dichotomized as Yes vs. No or ≥1 vs. 0 times/days. The denominator was all respondents for all outcomes *except* sexual dating violence, which was measured only among those who reported having dated someone in the past 12 months (56.9% and 46.7% of all respondents in 2019 and 2021, respectively). Table A in S1 Text provides survey questions, response options, and categorization for all outcomes.

### Analysis

It was possible to ascertain the predominant learning model for the 2020–2021 school year and obtain 2019 and 2021 YRBS data either in the public-use dataset or via request for 25 of 28 local jurisdictions. The remaining jurisdictions were excluded due to inability to ascertain learning model (n = 2) or obtain data (n = 1). We first assessed changes in outcomes from 2019 to 2021 in all included jurisdictions using Rao-Scott chi-square tests. Second, we used a DID approach to estimate relative changes in each outcome from 2019 to 2021 between jurisdictions where high school students primarily attended school virtually vs. in-person during the 2020–2021 school year. DID analysis is a quasi-experimental design commonly used to assess the impact of programs and practices [18]. It estimates the relative change as the difference of two differences, the difference in the outcome between the intervention and control groups and the difference in the outcome pre- and post-intervention within each group, thereby accounting for secular trends in the outcomes and time-invariant differences between the intervention and control groups. We calculated the percent of respondents reporting each outcome by learning model and survey year, absolute change in these percentages from 2019 to 2021 within each learning model, and–as an estimate of the DID–absolute difference in these two percentages (virtual minus in-person change). For statistical comparisons, we used logistic regression with the DID term parameterized as the interaction between survey year and learning model. When jurisdictions did not ask the question(s) for an outcome or its denominator in either 2019 or 2021, we excluded this jurisdiction from analyses for that outcome.

### Sensitivity analyses

We conducted two sets of sensitivity analyses. First, 15 (60%) of the 25 included jurisdictions changed from administering YRBS in spring semester in 2019 to fall semester in 2021, and this was more common among in-person (6/9 = 67%) than virtual (9/16 = 56%) jurisdictions [12]. Comparing 2019–2021, districts that changed the semester they administered YRBS had a different overall age distribution, age distribution within each grade, and calendar months in the recall period for outcomes measured in the past 30 days. We conducted analyses limited to the 10 jurisdictions that administered YRBS during the same semester in both years to estimate DIDs effects unaffected by these differential changes.

Second, the relationship between suicidal thoughts, making suicide plans, and attempting suicide is complex [19,20], and seriously considering suicide and having made a suicide plan can alert providers for the need for further intervention. Thus, we conducted additional analyses of suicide outcomes with the following alternative denominators: making a suicide plan among those who seriously considered attempting suicide, attempting suicide among those who made a suicide plan, and being injured in a suicide attempt among those who attempted suicide.

P-values<0.050 were considered statistically significant. All analyses accounted for complex survey design using survey procedures in SAS^®^ OnDemand for Academics.

## Results

### Study population

In the 25 included YRBS jurisdictions, there were a total of 49,740 respondents in 2019 and 42,995 respondents in 2021. In 16 jurisdictions comprising 64% of respondents, high school students predominantly attended school virtually during the 2020–2021 school year vs. in-person in 9 jurisdictions (Table 1). Virtual learning jurisdictions were distributed across all four census regions with large proportions of respondents from the Northeast (48.8–50.8%) and West (29.2–31.8%); all in-person jurisdictions were in the South. Approximately half of respondents across learning models and years reported female sex, and distributions of sexual orientation were similar across learning models (only available in 2021). While similar proportions reported being non-Hispanic Black across both years and learning models (26.0–27.5%), a greater proportion of respondents in virtual learning jurisdictions reported being Hispanic/Latino and Asian and greater proportions in in-person learning jurisdictions reported being non-Hispanic White.

### Overall changes from 2019 to 2021

Among respondents in all jurisdictions, reports of persistent feelings of sadness or hopelessness (2019 = 36.3%, 2021 = 41.3%), seriously considering attempting suicide (17.0–18.7%), and making a suicide plan (14.2–16.9%) increased significantly from 2019 to 2021, while suicide attempts and injuries during attempts did not significantly change (Table B in S1 Text). Alcohol use (22.5–17.1%), binge drinking alcohol (8.5–6.2%), and marijuana use (20.2–14.3%) in the past 30 days and ever prescription opioid misuse (16.0–13.2%) decreased significantly, and electronic vapor product use remained stable (14.8% in both years). Reports of carrying a gun (5.5–4.5%), being in a physical fight (22.3–17.3%), and experiencing any sexual violence (11.3–10.4%) in the past 12 months decreased significantly, whereas experiences of sexual dating violence increased significantly (6.7–7.8%) and electronic bullying remained stable (12.5–12.4%).

### Effects of school learning model

**Fig 1.**
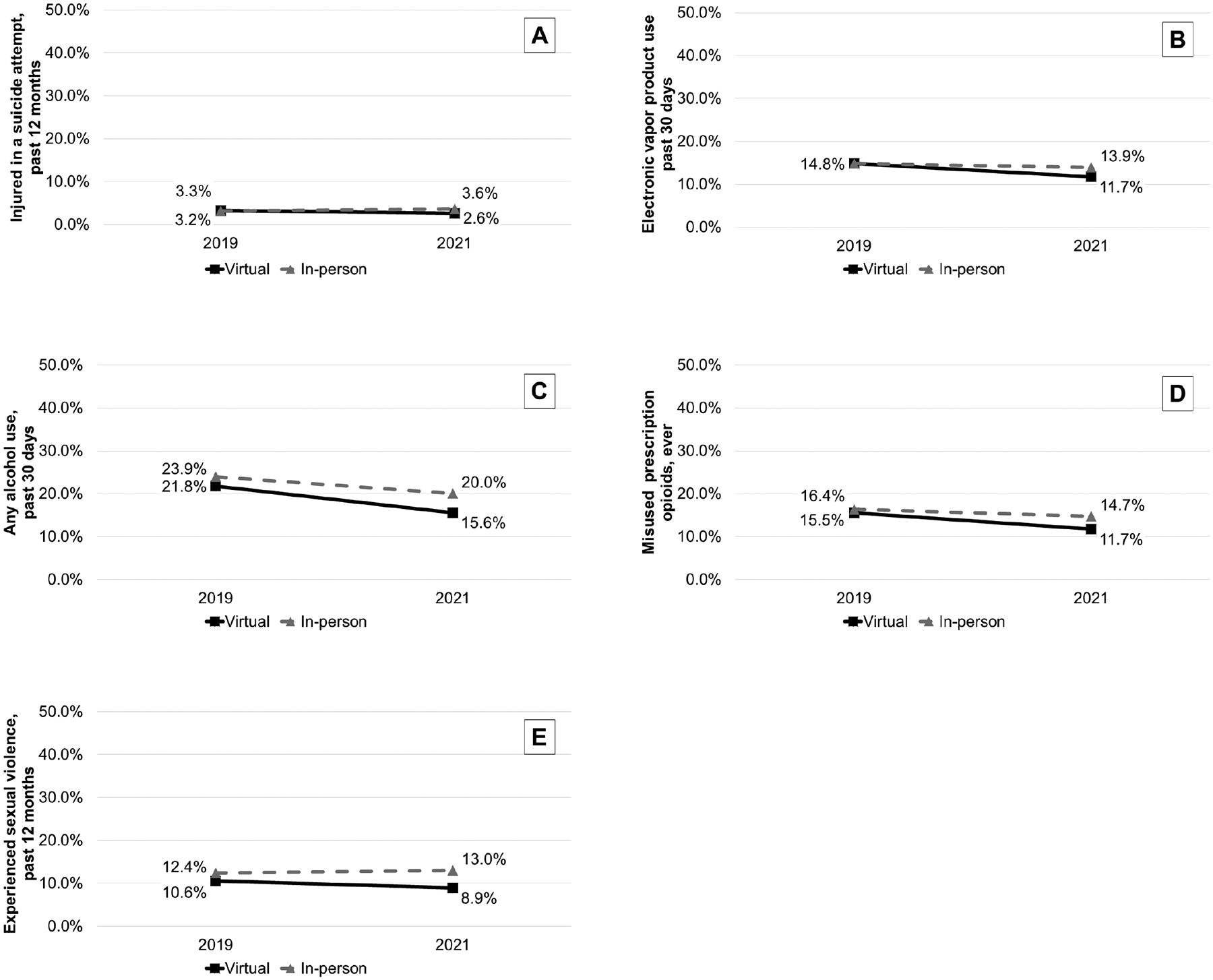
Estimated effects of virtual (vs. in-person) learning in 2020–2021 school year on being injured in a suicide attempt, electronic vapor product use, any alcohol use, misuse of prescription opioids, and experience of sexual violence. https://doi.org/10.1371/journal.pmen.0000409.g001

In main analyses, virtual learning was associated with relative decreases in being injured during a suicide attempt in the past 12 months [DID odds ratio (OR)=0.68, 95% confidence interval (CI)=0.52–0.88], electronic vapor product use in the past 30 days (OR=0.81, 95%CI = 0.70–0.95), any alcohol use in the past 30 days (OR=0.84, 95%CI = 0.74–0.95), ever misusing prescription opioids (OR=0.82, 95%CI = 0.72–0.94), and experiencing sexual violence in the past 12 months (OR=0.78, 95%CI = 0.67–0.91) [Tables 2 and 3; Fig 1]. Stratified by sex, these associations remained significant only among male respondents, except prescription opioid misuse which was significant for male and female respondents (Table 3; Table C in S1 Text). Additionally, virtual learning was associated with a relative increase in experiencing sexual dating violence among female respondents who reported dating in the past 12 months (OR=1.35, 95%CI = 1.02–1.79) and with relative decreases in marijuana use in the past 30 days (OR=0.82, 95%CI = 0.68–0.99), having been in a physical fight in the past 12 months (OR=0.80, 95%CI = 0.67–0.96), and having been electronically bullied in the past 12 months (OR=0.81, 95%CI = 0.66–1.00) among male respondents.

In jurisdictions (7 virtual, 3 in-person) that administered YRBS during the same semester in both years, DID estimates for attempting suicide, misuse of prescription opioids, being in a physical fight, and being electronically bullied were similar to overall estimates and represented similarly significant but stronger relative decreases associated with virtual learning for electronic vapor product use, any alcohol use, and experiencing any sexual violence (Table 3; Table D in S1 Text). New significant associations with virtual learning were identified: a relative increase in experiencing sexual dating violence and relative decreases in persistent feelings of sadness or hopelessness, seriously considering attempting suicide, making a suicide plan, and marijuana use. The association between virtual learning and being injured in a suicide attempt was no longer statistically significant.

In sensitivity analyses of suicide outcomes with alternate denominators, virtual learning remained associated with a relative decrease in being injured in a suicide attempt and not associated with the other suicide outcomes (Table E in S1 Text).

The assumption of parallel trends between virtual and in-person learning jurisdictions before the exposure appeared to hold for all outcomes except electronic vapor product use and, to a lesser extent, any alcohol use and sexual dating violence (Table F in S1 Text; S1 Fig).

## Discussion

In a population-based sample of high school students in 25 jurisdictions across the U.S., we found significant increases in several indicators of poor mental health and suicide, significant decreases in alcohol, marijuana, and prescription opioid misuse, and varied changes in violence related behaviors and experiences from 2019 to 2021. Jurisdictions with students attending school predominantly virtually in the 2020–2021 school year had relative decreases in being injured during a suicide attempt, electronic vapor product use, alcohol use, prescription opioid misuse, and experiencing sexual violence compared to jurisdictions with students attending school predominantly in-person. These relative decreases were observed primarily among male students, for whom relative decreases were also observed for marijuana use, being in a physical fight, and being bullied electronically. In contrast, virtual learning was associated with a relative increase in sexual dating violence among female students who had dated in the prior year.

Poor mental health and suicidal thoughts and behaviors among adolescents are a major public health concern, described by the American Academy of Pediatrics American Academy of Child and Adolescent Psychiatry, and Children’s Hospital Association as a national emergency [21]. Contrary to several prior studies [5,6,22], we found no association between virtual learning and worse indicators of poor mental health and suicide outcomes. Instead, virtual learning was associated with a relative decrease in injuries during suicide attempts in overall analyses and relative decreases in persistent feelings of sadness or hopelessness, seriously considering attempting suicide, and making a suicide plan in sensitivity analyses that accounted for potential bias from changes in semester of survey administration. For many youth, returning to classrooms full of people after spending substantial time at home and experiencing myriad challenges (e.g., death of loved ones, economic disruptions, loneliness) during the pandemic may have increased stress and contributed to poorer mental health and suicidal thoughts and behaviors [23]. Additionally, many prior studies assessed the impacts of school closures during the earliest phase of the pandemic, and the effects of longer-term virtual learning might differ, particularly if increasing opportunities for social connection as society began to open up mitigated some negative effects of virtual learning [24]. Differences in study design may also contribute to these differences in findings, and our population-level analysis may not reflect individual-level effects. The latter might be particularly relevant for adolescents who already experienced disparities in these outcomes, less access to mental health resources, and may have been more likely to experience COVID-19-related stressors [23,25–27]. Regardless, some adverse mental health and suicide outcomes continued to increase in both virtual and in-person learning jurisdictions, highlighting the need for new efforts to support youth mental health and suicide prevention efforts across all schools.

Experiences during the COVID-19 pandemic may have intensified risk factors for adolescent substance use, such as stress, social isolation, and boredom [28]. At the same time, stark reductions in opportunities for social contact may have resulted in reduced exposures, social pressures, and access to drugs, and changes in parental or school supervision could have mixed effects on substance use. Similar to national estimates [29,30], use of most substances decreased overall from 2019 to 2021 in our study jurisdictions and significantly more so in virtual than in-person learning jurisdictions, particularly for boys. This additional decrease associated with virtual learning is consistent with a previous study that found fewer students learning virtually (vs. in-person) had started or increased use of ≥1 substance to cope with stress or emotions [10]. Understanding how virtual learning might have yielded these relative reductions could inform development of interventions to reduce substance use.

Approximately 1 in 6 female and 1 in 12 male students reported experiencing sexual violence by anyone in the past year. Of those students who had dated, 1 in 10 female and 1 in 20 male students reported sexual dating violence in the past year. Overall, students in virtual learning jurisdictions reported decreases in experiencing any sexual violence from 2019 to 2021 while those in-person learning jurisdictions reported slight increases, particularly among male students. In contrast, experiences of sexual violence by a dating partner increased from 2019 to 2021 across learning model and sex, with a relatively greater increase in virtual learning jurisdictions, particularly among female students. Decreases in sexual violence experiences among male students in virtual learning jurisdictions may reflect an increased risk of sexual violence for boys in the school environment. For example, one study found that the most common form of sexual violence experienced by male high school students involved being “kissed, hugged, or sexually touched” by peers, with about 40% of those experiences happening at school [31]. However, girls experience much higher rates of sexual dating violence and forced sex than boys, experiences that may be less likely to occur on school grounds [32]. Pandemic-associated stress and isolation may have increased risk for mental health problems, a risk factor for dating violence [24,32,33]. Further, some youth in virtual learning environments (e.g., with parents working outside the home) may have experienced decreases in adult supervision, which could have increased their risk for experiencing sexual dating violence [34]. Additional efforts are needed to understand and identify opportunities to prevent exposure to sexual violence in schools among boys and relative increases in sexual dating violence among girls associated with virtual learning.

Our study design and data sources have several strengths. The quasi-experimental DID approach allows for causal inference and accounting for prior trends [18], and using YRBS data, a long-standing population-based survey, has potential to increase representativeness and limit bias from self- or proxy-perceptions of pandemic-related impacts. There are also important limitations. First, included jurisdictions are primarily urban and may not be representative of the entire U.S. However, our overall estimates and changes from 2019 to 2021 were generally similar to national YRBS estimates [29,30,32,35]. Second, jurisdictions that chose to continue virtual learning instead of returning to in-person learning at the beginning of the 2020–2021 school year are likely to have differed with respect to other COVID-related laws, policies, and community responses that may have contributed to or obscured differential changes in our outcomes. Of note, all in-person learning jurisdictions were within the South, mostly in Florida, whereas virtual learning jurisdictions represented a broader geographic range but were concentrated in the West and Northeast. In addition, when not all schools in a jurisdiction reported the same learning model for the entire period, summarizing our exposure across schools may have led to some misclassification. Fourth, data from prior YRBS survey years suggested that the assumption of parallel trends underlying DID analyses held for most, but not all, of our outcomes. Due to changes in survey questions, jurisdictions implementing YRBS, and jurisdiction-level decisions about which questions to include, we were unable to include all jurisdictions when testing this assumption or address the assumption for all outcomes. Additionally, outcomes were self-reported and therefore sensitive to social desirability and recall bias, and dichotomization may have masked changes in the frequency of some outcomes. Finally, recall periods in 2021 did not always overlap perfectly with the period of exposure to virtual vs. in-person learning, particularly among virtual learning jurisdictions, some of which returned to in-person learning as early as February 2021. Importantly, most associations in overall analyses remained significant when limiting analyses to jurisdictions that administered YRBS in the same semester in 2019 and 2021, though several new significant associations were identified, suggesting that changes in survey timing may have obscured some effects of learning models.

## Conclusions

Together, our findings highlight the importance of ensuring schools offer safe and supportive environments for all youth and promote equitable health and educational outcomes. A comprehensive, multidisciplinary and multi-level approach to school safety and climate is needed with attention to potential differences across learning models in student needs and opportunities for intervention, particularly as virtual learning environments continue to expand. Given documented relative increases in several adverse health behaviors and experiences in jurisdictions that returned to in-person learning at the start of the 2020–2021 school year, primarily among male students, providing additional supports when students return to in-person instruction following extended school closures or virtual learning might be needed. Notably, we observed continued, absolute increases in some adverse mental health and suicide outcomes in all jurisdictions, emphasizing the need to support youth mental health and suicide prevention efforts regardless of learning model. CDC recently created an action guide to help school and district leaders strengthen their work to promote students’ mental health and identify new strategies to address gaps, including providing social, emotional, and behavioral learning; psychosocial skills training; and cognitive behavioral interventions [36]. School-based health services, including behavioral health services, play an important role in improving access for adolescents [37], and finding approaches to maximize access to these services during emergency situations and their aftermath may be worthwhile. Addressing risk and protective factors for suicide, violence, and substance use by intervening at multiple levels of the social ecology may be most effective [38], and understanding how virtual learning might have yielded reductions in substance use and sex-specific changes in experiences of sexual violence relative to in-person learning could inform future programs and interventions for high school students. It will also be critical to examine whether virtual learning’s effects differed across factors other than sex, including multilevel factors influencing health and education, to help ensure future decisions regarding learning models do not worsen existing inequities.

## Supplementary Material

s1 S1 Table. School learning model dispositions data.
(XLSX)

s2 S1 Text. Tables A-F.
(DOCX)

s3 **S1 Fig. Supplemental Figure**. Test of parallel trend assumption - Trends in mental health, suicidal thoughts and behaviors, substance use, and violence related behaviors and experiences by school learning model in the 2020–2021 school year among Youth Risk Behavior Survey respondents in local jurisdictions with data from all survey years from 2013–2021.
(PPTX)

## Figures and Tables

**Table 1.** Sociodemographic characteristics of Youth Risk Behavior Survey respondents in 25 local jurisdictions, stratified by school learning model for the 2020–2021 school year and survey year. https://doi.org/10.1371/journal.pmen.0000409.t001

School learning model (2020–2021)	Virtual		In-person	
YRBS survey year	2019	2021	2019	2021
Unweighted N (% of total for year)	32358 (65.0%)	27058 (62.9%)	17382 (35.0%)	15937 (37.1%)
Sex				
Female	49.4%	48.8%	49.8%	49.4%
Male	50.6%	51.2%	50.2%	50.6%
Age				
≤12	0.6%	0.5%	0.8%	0.8%
13	1.6%	1.5%	0.4%	0.5%
14	17.6%	22.2%	11.3%	16.9%
15	25.2%	25.0%	24.8%	25.2%
16	24.6%	24.4%	24.5%	24.6%
17	21.5%	21.1%	24.4%	22.5%
≥18	9.0%	5.3%	13.8%	9.5%
Race and ethnicity				
American Indian/Alaska Native	1.1%	0.6%	0.7%	0.6%
Asian	10.1%	10.7%	4.0%	3.1%
Black/African American	26.0%	26.5%	27.3%	27.5%
Hispanic/Latino	43.4%	44.3%	36.4%	39.1%
Native Hawaiian/Pacific Islander	0.9%	0.3%	0.6%	0.4%
White	15.2%	14.9%	27.8%	26.3%
Multiple, Non-Hispanic	3.3%	2.7%	3.2%	3.0%
Sexual orientation*				
Heterosexual	–	71.3%	–	71.8%
Gay or lesbian	–	3.9%	–	5.2%
Bisexual	–	11.7%	–	11.7%
I describe my sexual identity some other way	–	4.5%	–	3.8%
I am not sure about my sexual identity	–	5.6%	–	5.0%
Don’t know what this question is asking	–	2.9%	–	2.4%
Census region				
Midwest	11.9%	12.4%	0%	0%
Northeast	48.8%	50.8%	0%	0%
South	7.5%	7.6%	100%	100%
West	31.8%	29.2%	0%	0%

YRBS = Youth Risk Behavioral Survey.

* Data were not available for 2019 in the combined YRBS dataset following changes in response options from the 2019 to 2021 survey. All analyses are survey-weighted. Virtual learning jurisdictions were: Los Angeles, CA; Oakland, CA; San Diego, CA; San Francisco, CA; Chicago, IL; Boston, MA; Gaston Co., NC; Newark, NJ; Albuquerque, NM; New York City, NY; Cleveland, OH; Portland, OR; Philadelphia, PA; Nashville, TN; Shelby Co., TN; Seattle, WA. In-person learning jurisdictions were: Broward Co, FL; Duval Co., FL; Hillsborough Co., FL; Orange Co., FL; Palm Beach Co., FL; Pasco Co., FL; Spartanburg Co., SC; Ft. Worth, TX; Houston, TX.

**Table 2.** Mental health, suicidal thoughts and behaviors, substance use, and violence related behaviors and experiences among Youth Risk Behavior Survey respondents, stratified by school learning model for the 2020–2021 school year and survey year. https://doi.org/10.1371/journal.pmen.0000409.t002

School learning model (2020–2021)	Virtual			In-person			Difference-in-Differences			
YRBS survey year	2019	2021	Difference	2019	2021	Difference	Absolute	OR	95%CI	p-value
*Poor Mental Health and Suicide, Past 12 Months*										
Persistent feelings of sadness or hopelessness	35.9%	40.4%	4.5%	37.0%	43.0%	6.0%	−1.5%	0.94	(0.84–1.05)	0.291
Seriously considered attempting suicide	16.2%	17.4%	1.2%	18.5%	21.1%	2.5%	−1.4%	0.93	(0.81–1.06)	0.266
Made a suicide plan	13.8%	16.0%	2.2%	14.7%	17.8%	3.1%	−1.0%	0.94	(0.84–1.06)	0.316
Attempted suicide	10.0%	9.7%	−0.3%	11.4%	11.5%	0.1%	−0.4%	0.96	(0.80–1.14)	0.609
Injured in a suicide attempt	3.3%	2.6%	−0.8%	3.2%	3.6%	0.4%	−1.2%	0.68	(0.52–0.88)	0.004
*Substance Use*										
Electronic vapor product, past 30 days	14.8%	11.7%	−3.1%	14.8%	13.9%	−0.8%	−2.3%	0.81	(0.70–0.95)	0.009
Alcohol - any use, past 30 days	21.8%	15.6%	−6.1%	23.9%	20.0%	−4.0%	−2.2%	0.84	(0.74–0.95)	0.006
Alcohol - binge drinking, past 30 days	8.4%	6.0%	−2.4%	8.6%	6.6%	−1.9%	−0.4%	0.92	(0.76–1.12)	0.415
Marijuana, past 30 days	20.6%	14.1%	−6.6%	19.3%	14.6%	−4.7%	−1.9%	0.88	(0.75–1.04)	0.132
Misused prescription opioids, ever	15.5%	11.7%	−3.8%	16.4%	14.7%	−1.7%	−2.2%	0.82	(0.72–0.94)	0.003
*Violence Related Behaviors and Experiences, Past 12 Months*										
Carried a gun	4.9%	3.9%	−1.0%	5.9%	4.9%	−1.0%	0.0%	0.96	(0.74–1.26)	0.788
In a physical fight	22.7%	17.0%	−5.7%	21.5%	17.8%	−3.7%	−2.0%	0.88	(0.75–1.03)	0.123
Experienced sexual violence	10.6%	8.9%	−1.7%	12.4%	13.0%	0.6%	−2.3%	0.78	(0.67–0.91)	0.002
Experienced sexual dating violence*	6.3%	7.9%	1.6%	7.4%	7.7%	0.3%	1.3%	1.22	(0.96–1.56)	0.096
Electronically bullied	12.3%	12.1%	−0.2%	12.8%	12.8%	0.0%	−0.2%	0.98	(0.85–1.13)	0.773

OR = odds ratio. 95%CI = 95% confidence interval. All analyses are survey-weighted.

* Measured among respondents reporting dating or going out with someone in the past 12 months; all other outcomes measured among all respondents. For the following outcomes, jurisdictions in parentheses did not have data in the public-use dataset for the numerator or denominator and were excluded from analyses for that outcome: Seriously considered attempting suicide (Spartanburg Co., SC); Made a suicide plan (New York City, NY; San Diego, CA); Attempted suicide (Spartanburg Co., SC); Injured in a suicide attempt (Cleveland, OH; Duval Co., FL; Gaston Co., NC; Shelby Co., TN); Alcohol – binge drinking (Oakland, CA; Shelby Co., TN; Spartanburg Co., SC); Misused prescription opioids (New York City, NY; Oakland, CA; Seattle, WA); Carried a gun (Boston, MA; Cleveland, OH; Chicago, IL; Duval Co., FL; Nashville, TN; New York City, NY; Portland, OR; Seattle, WA; San Francisco, CA; Spartanburg Co., SC); In a physical fight (Duval Co., FL; San Diego, CA; Seattle, WA); Experienced sexual violence (Boston, MA; Cleveland, OH; San Diego, CA; Spartanburg, SC); Experienced sexual dating violence (Albuquerque, NM; Boston, MA; Gaston Co., NC).

**Table 3.** Difference-in-differences estimates of the effect of virtual (vs. in-person) learning in the 2020–2021 school year on mental health, suicidal thoughts and behaviors, substance use, and violence related behaviors and experiences among Youth Risk Behavior Survey respondents. https://doi.org/10.1371/journal.pmen.0000409.t003

Outcomes	Difference-in-Differences Odds Ratio (95% Confidence interval)			
	Overall	Sex		Administered Survey in Same Semester
		Female	Male	
*Poor Mental Health and Suicide, Past 12 Months*				
Persistent feelings of sadness or hopelessness	0.94 (0.84–1.05)	0.97 (0.85–1.09)	0.91 (0.78–1.06)	0.81 (0.69–0.95)*
Seriously considered attempting suicide	0.93 (0.81–1.06)	0.97 (0.83–1.13)	0.86 (0.72–1.04)	0.78 (0.64–0.96)*
Made a suicide plan	0.94 (0.84–1.06)	1.01 (0.86–1.17)	0.87 (0.72–1.05)	0.73 (0.61–0.87)*
Attempted suicide	0.96 (0.80–1.14)	1.02 (0.84–1.24)	0.84 (0.65–1.09)	1.00 (0.76–1.32)
Injured in a suicide attempt	0.68 (0.52–0.88)*	0.79 (0.56–1.10)	0.51 (0.33–0.79)*	0.89 (0.55–1.44)
*Substance Use*				
Electronic vapor product, past 30 days	0.81 (0.70–0.95)*	0.90 (0.72–1.12)	0.71 (0.58–0.87)*	0.64 (0.50–0.82)*
Alcohol - any use, past 30 days	0.84 (0.74–0.95)	0.89 (0.75–1.06)	0.77 (0.67–0.89)	0.65 (0.52–0.80)*
Alcohol - binge drinking, past 30 days	0.92 (0.76–1.12)	0.96 (0.75–1.23)	0.86 (0.65–1.13)	0.77 (0.57–1.03)
Marijuana, past 30 days	0.88 (0.75–1.04)	0.93 (0.76–1.14)	0.82 (0.68–0.99)*	0.71 (0.56–0.90)*
Misused prescription opioids, ever	0.82 (0.72–0.94)*	0.83 (0.71–0.98)*	0.80 (0.66–0.97)*	0.87 (0.73–1.04)
*Violence Related Behaviors and Experiences, Past 12 Months*				
Carried a gun	0.96 (0.74–1.26)	1.00 (0.60–1.64)	0.95 (0.74–1.21)	0.68 (0.46–1.00)
In a physical fight	0.88 (0.75–1.03)	0.99 (0.80–1.23)	0.80 (0.67–0.96)*	0.89 (0.67–1.19)
Experienced sexual violence	0.78 (0.67–0.91)*	0.89 (0.75–1.04)	0.63 (0.49–0.82)*	0.64 (0.51–0.82)*
Experienced sexual dating violence^	1.22 (0.96–1.56)	1.35 (1.02–1.79)*	1.25 (0.83–1.89)	1.68 (1.17–2.40)*
Electronically bullied	0.98 (0.85–1.13)	1.11 (0.93–1.32)	0.81 (0.66–1.00)*	0.90 (0.72–1.13)

All estimates are from survey-weighted logistic regression. Difference-in-differences estimates from the interaction term between survey year (2019 vs. 2021) and learning model (virtual vs. in-person).

* p < 0.050.

^ Measured among respondents reporting dating or going out with someone in the past 12 months; all other outcomes measured among all respondents.

## Data Availability

Data for this study are publicly available as part of the 2021 Youth Risk Behavior Survey public-use dataset (https://www.cdc.gov/yrbs/data/index.html), upon request from local health or education departments participating in the Youth Risk Behavior Survey, and from the COVID-19 School Data Hub (https://www.covidschooldatahub.com/).
