# Supplementary material for: Impact of COVID-19 school learning model on mental health, suicidal thoughts and behaviors, substance use, and violence related behaviors and experiences among U.S. high school students
Source: PLOS Ment Health. 2025 Sep 17;2(9):e0000409. doi: 10.1371/journal.pmen.0000409 (PMC12724680; doi:10.1371/journal.pmen.0000409)
Supplement: S1 Text — (DOCX) [file pmen.0000409.s002.docx]

**S1 Text – Tables A-F for Katz et al. Impact of COVID-19 School Learning Model on Mental Health, Suicidal Thoughts and Behaviors, Substance Use, and Violence Related Behaviors and Experiences Among U.S. High School Students**

| **Table A. Outcome definition for poor mental health, suicidal thoughts and behaviors, substance use, and violence related behaviors and experiences in Youth Risk Behavior Survey, 2019 and 2021** | | | |
| --- | --- | --- | --- |
| **Outcome** | **Survey Question** | **Response Options** | **Dichotomization** |
| *Poor Mental Health and Suicide, Past 12 Months* |  |  |  |
| Persistent feelings of sadness or hopelessness | During the past 12 months, did you ever feel so sad or hopeless almost every day for two weeks or more in a row that you stopped doing some usual activities? | Yes  No | Yes vs. No |
| Seriously considered attempting suicide | During the past 12 months, did you ever seriously consider attempting suicide? | Yes  No | Yes vs. No |
| Made a suicide plan | During the past 12 months, did you make a plan about how you would attempt suicide? | Yes  No | Yes vs. No |
| Attempted suicide | During the past 12 months, how many times did you actually attempt suicide? | 0 times, 1 time, 2 or 3 times, 4 or 5 times,  6 or more times | ≥1 vs. 0 times |
| Injured in a suicide attempt | If you attempted suicide during the past 12 months, did any attempt result in an injury, poisoning, or overdose that had to be treated by a doctor or nurse? | I did not attempt suicide during the past 12 months; Yes; No | Yes vs. No or I did not attempt suicide |
|  |  |  |  |
| *Substance Use* |  |  |  |
| Electronic vapor product, past 30 days | During the past 30 days, on how many days did you use an electronic vapor product? | 0 days, 1 or 2 days. 3 to 5 days. 6 to 9 days. 10 to 19 days. 20 to 29 days. All 30 days | ≥1 vs. 0 days |
| Alcohol - any use, past 30 days | During the past 30 days, on how many days did you have at least one drink of alcohol? | 0 days, 1 or 2 days  3 to 5 days, 6 to 9 days, 10 to 19 days  20 to 29 days, All 30 days | ≥1 vs. 0 days |
| Alcohol - binge drinking, past 30 days | During the past 30 days, on how many days did you have 4 or more drinks of alcohol in a row, that is, within a couple of hours (if you are female) or 5 or more drinks of alcohol in a row, that is, within a couple of hours (if you are male)? | 0 days, 1 day  2 days, 3 to 5 days  6 to 9 days,  10 to 19 days, More than 20 days, All 30 days | ≥1 vs. 0 days |
| Marijuana, past 30 days | During the past 30 days, how many times did you use marijuana? | 0 times, 1 to 2 times  3 to 9 times. 10 to 19 times, 20 to 39 times, 40 or more times | ≥1 vs. 0 times |
| Misused prescription opioids, ever | During your life, how many times have you taken prescription pain medicine without a doctor's prescription or differently than how a doctor told you to use it? | 0 times, 1 to 2 times, 3 to 9 times, 10 to 19 times, 20 to 39 times, 40 or more times | ≥1 vs. 0 times |
|  |  |  |  |
| *Violence Related Behaviors and Experiences, Past 12 Months* |  |  |  |
| Carried a gun | During the past 12 months, on how many days did you carry a gun? (Do not count the days when you carried a gun only for hunting or for a sport, such as target shooting.) | 0 days, 1 day, 2 or 3 days, 4 or 5 days, 6 or more days | ≥1 vs. 0 days |
| In a physical fight | During the past 12 months, how many times were you in a physical fight? | 0 times, 1 time, 2 or 3 times, 4 or 5 times, 6 or 7 times, 8 or 9 times, 10 or 11 times, 12 or more times | ≥1 vs. 0 times |
| Experienced sexual violence | During the past 12 months, how many times did anyone force you to do sexual things that you did not want to do? (Count such things as kissing, touching, or being physically forced to have sexual intercourse.) | 0 times, 1 time, 2 or 3 times, 4 or 5 times, 6 or more times | ≥1 vs. 0 times |
| Experienced sexual dating violence | During the past 12 months, how many times did someone you were dating or going out with force you to do sexual things that you did not want to do? (Count such things as kissing, touching, or being physically forced to have sexual intercourse.) | I did not date or go out with anyone in the past 12 months, 0 times, 1 time, 2 or 3 times, 4 or 5 times, 6 or more times | ≥1 vs. 0 times (excluded respondents who did not date or go out with anyone) |
| Electronically bullied | During the past 12 months, have you ever been electronically bullied? (Count being bullied through texting, Instagram, Facebook, or other social media.) | Yes  No | Yes vs. No |

| **Table B. Mental health, suicidal thoughts and behaviors, substance use, and violence related behaviors and experiences among Youth Risk Behavior Survey respondents, stratified by survey year** | | | | | |
| --- | --- | --- | --- | --- | --- |
| **YRBS Survey Year** | **2019**  **% (95%CI)** | **2021**  **% (95%CI)** | **Difference** | **p-value*** |  |
| *Poor Mental Health and Suicide, Past 12 Months* |  |  |  |  |  |
| Persistent feelings of sadness or hopelessness | 36.3% (35.5-37.0%) | 41.3% (40.2-42.4%) | 5.0% | <0.0001 |  |
| Seriously considered attempting suicide | 17.0% (16.4-17.6%) | 18.7% (17.9-19.4%) | 1.7% | 0.001 |  |
| Made a suicide plan | 14.2% (13.7-14.8%) | 16.9% (16.3-17.5%) | 2.7% | <0.0001 |  |
| Attempted suicide | 10.5% (9.9-11.1%) | 10.3% (9.7-10.9%) | -0.2% | 0.64 |  |
| Injured in a suicide attempt | 3.3% (3.0-3.6%) | 2.9% (2.6-3.2%) | -0.4% | 0.07 |  |
|  |  |  |  |  |  |
| *Substance Use* |  |  |  |  |  |
| Electronic vapor product, past 30 days | 14.8% (13.6-16.0%) | 14.8% (14.0-15.6%) | 0.0% | 0.96 |  |
| Alcohol - any use, past 30 days | 22.5% (21.7-23.4%) | 17.1% (16.0-18.2%) | -5.4% | <0.0001 |  |
| Alcohol - binge drinking, past 30 days | 8.5% (7.9-9.0%) | 6.2% (5.7-6.8%) | -2.2% | <0.0001 |  |
| Marijuana, past 30 days | 20.2% (19.5-20.8%) | 14.3% (13.3-15.3%) | -5.9% | <0.0001 |  |
| Misused prescription opioids, ever | 16.0% (15.3-16.6%) | 13.2% (12.6-13.8%) | -2.8% | <0.0001 |  |
|  |  |  |  |  |  |
| *Violence Related Behaviors and Experiences, Past 12 Months* |  |  |  |  |  |
| Carried a gun | 5.5% (4.9-6.0%) | 4.5% (4.1-4.9%) | -1.0% | 0.004 |  |
| In a physical fight | 22.3% (21.4-23.1%) | 17.3% (16.2-18.3%) | -5.0% | <0.0001 |  |
| Experienced sexual violence | 11.3% (10.8-11.8%) | 10.4% (9.8-11.0%) | -0.8% | 0.04 |  |
| Experienced sexual dating violence**^†^** | 6.7% (6.3-7.1%) | 7.8% (7.1-8.5%) | 1.1% | 0.01 |  |
| Electronically bullied | 12.5% (11.9-13.1%) | 12.4% (11.8-12.9%) | -0.1% | 0.75 |  |
| YRBS = Youth Risk Behavior Survey. *From Rao-Scott chi-square tests to account for complex survey design. **^†^**Measured among respondents reporting dating or going out with someone in the past 12 months; all other outcomes measured among all respondents. | | | | | |

| **Table C. Mental health, suicidal thoughts and behaviors, substance use, and violence related behaviors and experiences among Youth Risk Behavior Survey respondents by school learning model for the 2020-2021 school year and survey year, stratified by sex** | | | | | | | | | | | |
| --- | --- | --- | --- | --- | --- | --- | --- | --- | --- | --- | --- |
| **School Learning Model (2020-2021)** |  | **Virtual** | | | **In-person** | | | **Difference-in-Differences (DID)** | | | |
| **YRBS Survey Year** |  | **2019** | **2021** | **Difference** | **2019** | **2021** | **Difference** | **Absolute** | **OR** | **95%CI** | **p-value** |
| *Poor Mental Health and Suicide, Past 12 Months* | **Sex** |  |  |  |  |  |  |  |  |  |  |
| Persistent feelings of sadness or hopelessness | Female | 44.4% | 52.1% | 7.7% | 46.3% | 54.9% | 8.6% | -0.9% | 0.97 | (0.85-1.09) | 0.586 |
|  | Male | 27.3% | 28.8% | 1.5% | 27.3% | 30.7% | 3.4% | -2.0% | 0.91 | (0.78-1.06) | 0.239 |
| Seriously considered attempting suicide | Female | 19.6% | 23.2% | 3.6% | 22.9% | 27.6% | 4.7% | -1.0% | 0.97 | (0.83-1.13) | 0.680 |
|  | Male | 12.5% | 11.2% | -1.4% | 13.9% | 14.1% | 0.2% | -1.6% | 0.86 | (0.72-1.04) | 0.113 |
| Made a suicide plan | Female | 16.9% | 21.1% | 4.2% | 18.4% | 22.8% | 4.4% | -0.2% | 1.01 | (0.86-1.17) | 0.940 |
|  | Male | 10.3% | 10.7% | 0.4% | 10.6% | 12.4% | 1.8% | -1.4% | 0.87 | (0.72-1.05) | 0.155 |
| Attempted suicide | Female | 10.5% | 11.7% | 1.2% | 12.1% | 13.3% | 1.1% | 0.1% | 1.02 | (0.84-1.24) | 0.821 |
|  | Male | 8.9% | 7.0% | -1.9% | 9.6% | 8.9% | -0.7% | -1.2% | 0.84 | (0.65-1.09) | 0.184 |
| Injured in a suicide attempt | Female | 3.1% | 2.9% | -0.2% | 3.3% | 3.9% | 0.6% | -0.8% | 0.79 | (0.56-1.10) | 0.163 |
|  | Male | 3.3% | 1.9% | -1.4% | 2.7% | 3.1% | 0.4% | -1.7% | **0.51** | **(0.33-0.79)** | **0.003** |
|  |  |  |  |  |  |  |  |  |  |  |  |
| *Substance Use* |  |  |  |  |  |  |  |  |  |  |  |
| Electronic vapor product, past 30 days | Female | 14.9% | 14.5% | -0.3% | 14.4% | 15.4% | 1.0% | -1.4% | 0.90 | (0.72-1.12) | 0.339 |
|  | Male | 14.5% | 8.6% | -5.9% | 14.6% | 11.8% | -2.8% | -3.1% | **0.71** | **(0.58-0.87)** | **0.0008** |
| Alcohol - any use, past 30 days | Female | 23.8% | 18.4% | -5.5% | 26.9% | 22.9% | -4.0% | -1.4% | 0.89 | (0.75-1.06) | 0.206 |
|  | Male | 19.3% | 12.7% | -6.6% | 20.4% | 16.8% | -3.6% | -3.1% | **0.77** | **(0.67-0.89)** | **0.0003** |
| Alcohol - binge drinking, past 30 days | Female | 8.9% | 6.6% | -2.2% | 9.7% | 7.5% | -2.1% | -0.1% | 0.96 | (0.75-1.23) | 0.753 |
|  | Male | 7.7% | 5.1% | -2.6% | 7.3% | 5.6% | -1.7% | -0.9% | 0.86 | (0.65-1.13) | 0.274 |
| Marijuana, past 30 days | Female | 21.5% | 16.3% | -5.3% | 19.7% | 15.7% | -4.0% | -1.3% | 0.93 | (0.76-1.14) | 0.498 |
|  | Male | 19.4% | 11.7% | -7.8% | 18.6% | 13.2% | -5.4% | -2.4% | **0.82** | **(0.68-0.99)** | **0.044** |
| Misused prescription opioids, ever | Female | 16.2% | 13.8% | -2.4% | 16.8% | 16.7% | -0.1% | -2.3% | **0.83** | **(0.71-0.98)** | **0.024** |
|  | Male | 14.4% | 9.4% | -4.9% | 15.0% | 12.1% | -2.9% | -2.0% | **0.80** | **(0.66-0.97)** | **0.023** |
|  |  |  |  |  |  |  |  |  |  |  |  |
| *Violence and Bullying, Past 12 Months* |  |  |  |  |  |  |  |  |  |  |  |
| Carried a gun | Female | 2.3% | 2.0% | -0.3% | 2.8% | 2.5% | -0.3% | 0.1% | 1.00 | (0.60-1.64) | 0.988 |
|  | Male | 7.1% | 5.5% | -1.6% | 8.1% | 6.6% | -1.4% | -0.1% | 0.95 | (0.74-1.21) | 0.669 |
| In a physical fight | Female | 17.3% | 13.2% | -4.2% | 16.4% | 12.5% | -3.9% | -0.3% | 0.99 | (0.80-1.23) | 0.942 |
|  | Male | 27.4% | 20.1% | -7.4% | 26.1% | 22.6% | -3.5% | -3.9% | **0.80** | **(0.67-0.96)** | **0.019** |
| Experienced sexual violence | Female | 12.4% | 12.5% | 0.2% | 16.0% | 17.8% | 1.9% | -1.7% | 0.89 | (0.75-1.04) | 0.149 |
|  | Male | 8.3% | 5.2% | -3.1% | 8.1% | 7.8% | -0.3% | -2.8% | **0.63** | **(0.49-0.82)** | **0.0005** |
| Experienced sexual dating violence* | Female | 7.5% | 11.3% | 3.8% | 10.0% | 11.4% | 1.4% | 2.4% | 1.35 | (1.02-1.79) | 0.037 |
|  | Male | 4.2% | 4.2% | 0.0% | 4.2% | 3.4% | -0.9% | 0.8% | 1.25 | (0.83-1.89) | 0.288 |
| Electronically bullied | Female | 13.8% | 14.3% | 0.6% | 15.5% | 14.7% | -0.7% | 1.3% | 1.11 | (0.93-1.32) | 0.242 |
|  | Male | 10.9% | 9.8% | -1.1% | 9.7% | 10.5% | 0.8% | -1.9% | **0.81** | **(0.66-1.00)** | **0.045** |
| YRBS = Youth Risk Behavior Survey. OR = odds ratio. 95%CI = 95% confidence interval. DID estimates represent the interaction term between survey year (2019 vs. 2021) and learning model (virtual vs. in-person) in logistic regression models. All analyses use survey analysis procedures to account for complex sample design. *Measured among respondents reporting dating or going out with someone in the past 12 months; all other outcomes measured among all respondents. | | | | | | | | | | | |

| **Table D. Mental health, suicidal thoughts and behaviors, substance use, and violence related behaviors and experiences among Youth Risk Behavior Survey respondents among jurisdictions that administered the Youth Risk Behavior Survey during the same semester in the 2019 and 2021 survey years, stratified by school learning model for the 2020-2021 school year and survey year** | | | | | | | | | | | |
| --- | --- | --- | --- | --- | --- | --- | --- | --- | --- | --- | --- |
| **School Learning Model (2020-2021)** | **Virtual** | | | **In-person** | | | **Difference-in-Differences** | | | |  |
| **YRBS Survey Year** | **2019** | **2021** | **Difference** | **2019** | **2021** | **Difference** | **Absolute** | **OR** | **95%CI** | **p-value** | |
| *Poor Mental Health and Suicide, Past 12 Months* |  |  |  |  |  |  |  |  |  |  | |
| Persistent feelings of sadness or hopelessness | 35.7% | 38.6% | 2.9% | 39.0% | 47.2% | 8.3% | -5.3% | **0.81** | **(0.69-0.95)** | **0.011** | |
| Seriously considered attempting suicide | 16.1% | 16.3% | 0.2% | 20.4% | 24.9% | 4.5% | -4.3% | **0.78** | **(0.64-0.96)** | **0.019** | |
| Made a suicide plan | 15.1% | 15.3% | 0.2% | 15.5% | 20.5% | 5.0% | -4.8% | **0.73** | **(0.61-0.87)** | **0.0005** | |
| Attempted suicide | 9.5% | 9.2% | -0.3% | 13.3% | 12.8% | -0.5% | 0.1% | 1.00 | (0.76-1.32) | 0.982 | |
| Injured in a suicide attempt | 3.1% | 2.4% | -0.7% | 4.0% | 3.5% | -0.5% | -0.2% | 0.89 | (0.55-1.44) | 0.641 | |
|  |  |  |  |  |  |  |  |  |  |  | |
| *Substance Use* |  |  |  |  |  |  |  |  |  |  | |
| Electronic vapor product, past 30 days | 16.5% | 12.0% | -4.5% | 16.9% | 18.0% | 1.1% | -5.6% | **0.64** | **(0.50-0.82)** | **0.0004** | |
| Alcohol - any use, past 30 days | 21.9% | 14.5% | -7.4% | 24.2% | 23.0% | -1.2% | -6.2% | **0.65** | **(0.52-0.80)** | **<0.0001** | |
| Alcohol - binge drinking, past 30 days | 9.4% | 5.9% | -3.6% | 10.4% | 8.3% | -2.1% | -1.5% | 0.77 | (0.57-1.03) | 0.081 | |
| Marijuana, past 30 days | 19.1% | 12.9% | -6.2% | 21.6% | 19.6% | -2.0% | -4.2% | **0.71** | **(0.56-0.90)** | **0.005** | |
| Misused prescription opioids, ever | 15.3% | 12.4% | -2.9% | 17.8% | 16.3% | -1.5% | -1.4% | 0.87 | (0.73-1.04) | 0.132 | |
|  |  |  |  |  |  |  |  |  |  |  | |
| *Violence Related Behaviors and Experiences, Past 12 Months* |  |  |  |  |  |  |  |  |  |  | |
| Carried a gun | 6.7% | 4.0% | -2.7% | 5.7% | 4.9% | -0.8% | -1.9% | 0.68 | (0.46-1.00) | 0.052 | |
| In a physical fight | 22.8% | 17.0% | -5.8% | 22.8% | 18.7% | -4.2% | -1.7% | 0.89 | (0.67-1.19) | 0.433 | |
| Experienced sexual violence | 10.2% | 8.6% | -1.6% | 13.4% | 16.5% | 3.1% | -4.8% | **0.64** | **(0.51-0.82)** | **0.0003** | |
| Experienced sexual dating violence* | 6.6% | 8.8% | 2.2% | 9.1% | 7.8% | -1.3% | 3.5% | **1.68** | **(1.17-2.40)** | **0.005** | |
| Electronically bullied | 14.1% | 13.0% | -1.1% | 14.0% | 14.3% | 0.3% | -1.4% | 0.90 | (0.72-1.13) | 0.383 | |
| YRBS = Youth Risk Behavior Survey. OR = odds ratio. 95%CI = 95% confidence interval. All analyses are survey-weighted. Bold indicates significance at p<0.050. *Measured among respondents reporting dating or going out with someone in the past 12 months; all other outcomes measured among all respondents. *Includes the following jurisdictions that administered YRBS in the same semester (either spring or fall) in both periods: Virtual = Albuquerque, NM; Cleveland, OH; Newark, NJ; New York City, NY; Portland, OR; San Francisco, CA; Seattle, WA; In-person = Duval County, FL; Hillsborough County, FL; Pasco County, FL. | | | | | | | | | | | |

| **Table E. Suicide outcomes among Youth Risk Behavior Survey respondents with sequential denominators, stratified by school learning model for the 2020-2021 school year and survey year** | | | | | | | | | | | |
| --- | --- | --- | --- | --- | --- | --- | --- | --- | --- | --- | --- |
| **School Learning Model (2020-2021)** | **Denominator** | **Virtual** | | | **In-person** | | | **Difference-in-Differences** | | | |
| **YRBS Survey Year** |  | **2019** | **2021** | **Difference** | **2019** | **2021** | **Difference** | **Absolute** | **OR** | **95%CI** | **p-value** |
| Seriously considered attempting suicide | All | 16.2% | 17.4% | 1.2% | 18.5% | 21.1% | 2.5% | -1.4% | 0.93 | (0.81-1.06) | 0.266 |
| Made a suicide plan | Seriously considered attempting suicide | 60.1% | 64.5% | 4.5% | 59.4% | 62.6% | 3.2% | 1.3% | 1.06 | (0.86-1.30) | 0.586 |
| Attempted suicide | Made a suicide plan | 44.4% | 41.3% | -3.1% | 42.6% | 43.1% | 0.4% | -3.5% | 0.87 | (0.70-1.07) | 0.188 |
| Injured in a suicide attempt | Attempted suicide | 35.9% | 28.2% | -7.7% | 31.7% | 32.1% | 0.4% | -8.1% | 0.68 | (0.52-0.88) | 0.004 |
| YRBS = Youth Risk Behavior Survey. OR = odds ratio. 95%CI = 95% confidence interval. All analyses are survey-weighted. Bold indicates significance at p<0.050. | | | | | | | | | | | |

| **Table F. Mental health, suicidal thoughts and behaviors, substance use, and violence related behaviors and experiences among Youth Risk Behavior Survey respondents by survey year and school learning model for the 2020-2021 school year, 2013-2021 (test of parallel trends assumption)** | | | | | | | | | | |
| --- | --- | --- | --- | --- | --- | --- | --- | --- | --- | --- |
| **Survey year** | **2013** | | **2015** | | **2017** | | **2019** | | **2021** | |
| **Outcome (number of virtual / number of in-person jurisdictions)** | **Virtual** | **In-person** | **Virtual** | **In-person** | **Virtual** | **In-person** | **Virtual** | **In-person** | **Virtual** | **In-person** |
| *Poor Mental Health and Suicide, Past 12 Months* |  |  |  |  |  |  |  |  |  |  |
| Persistent feelings of sadness or hopelessness (6 / 5) | 28.0% | 27.5% | 29.5% | 30.2% | 31.3% | 31.2% | 35.6% | 36.8% | 39.6% | 41.8% |
| Seriously considered attempting suicide (6 / 5) | 13.3% | 14.8% | 13.8% | 16.2% | 15.1% | 16.0% | 15.6% | 18.5% | 16.9% | 20.4% |
| Made a suicide plan (4 / 5) | 12.1% | 13.4% | 12.7% | 14.4% | 11.7% | 14.3% | 12.4% | 15.0% | 16.2% | 17.4% |
| Attempted suicide (6 / 5) | 8.4% | 9.4% | 8.6% | 11.4% | 9.8% | 11.5% | 9.5% | 11.7% | 9.5% | 11.8% |
| Injured in a suicide attempt (6 / 4) | 2.8% | 3.8% | 2.6% | 3.8% | 3.1% | 3.6% | 3.2% | 2.8% | 2.7% | 3.7% |
|  |  |  |  |  |  |  |  |  |  |  |
| *Substance Use* |  |  |  |  |  |  |  |  |  |  |
| Electronic vapor product, past 30 days* (8 / 6) | n/a | n/a | 16.5% | 22.1% | 12.6% | 8.5% | 13.7% | 13.9% | 10.9% | 12.9% |
| Alcohol - any use, past 30 days (6 / 5) | 26.4% | 32.7% | 21.7% | 30.2% | 19.6% | 27.9% | 20.9% | 24.1% | 14.4% | 19.3% |
| Marijuana, past 30 days (6 / 5) | 18.7% | 23.3% | 16.8% | 21.9% | 17.5% | 19.7% | 19.3% | 19.1% | 12.3% | 13.7% |
|  |  |  |  |  |  |  |  |  |  |  |
| *Violence Related Behaviors and Experiences, Past 12 Months* |  |  |  |  |  |  |  |  |  |  |
| In a physical fight (5 / 4) | 25.0% | 23.7% | 21.4% | 21.8% | 22.5% | 21.6% | 22.1% | 21.0% | 16.4% | 17.7% |
| Experienced sexual dating violence† (4 / 4) | 11.0% | 10.8% | 10.5% | 10.6% | 12.3% | 6.8% | 6.4% | 6.5% | 8.6% | 7.3% |
| Electronically bullied (6 / 5) | 10.1% | 11.1% | 11.2% | 12.7% | 12.3% | 12.9% | 12.4% | 12.7% | 12.5% | 12.5% |
| YRBS = Youth Risk Behavior Survey. Analyses conducted in up to 11 jurisdictions with data from all survey years 2013-21 (of 25 total), comprising 50862/92735 (54.9%) of total respondents in overall difference-in-differences analyses. Includes: Los Angeles, CA; New York City, NY; Philadelphia, PA; San Diego, CA; San Francisco, CA; Broward Co., FL; Duval Co., FL; Orange Co., FL; Palm Beach Co., FL; Boston, MA; Houston, TX. *Electronic vapor product use was not asked in 2013 and therefore analyzed in 2015-2021 only with 3 additional jurisdictions as described below. †Measured among respondents reporting dating or going out with someone in the past 12 months; all other outcomes measured among all respondents. Conducting these analyses using data from 2015-2021 only, which allowed the inclusion of up to 3 additional jurisdictions (Cleveland, OH; Oakland, CA; Ft. Worth, TX) and comprised 59472/92735 (64.1%) of respondents included in the difference-in-differences analyses, yielded similar results. Similar analyses of binging alcohol, prescription opioid misuse, carrying a gun, and experiencing any sexual violence were not possible because these questions were either not asked or only asked by 1 jurisdiction in 2013 and 2015. | | | | | | | | | | |
