# Supplementary material for: Impact of COVID-19 school learning model on mental health, suicidal thoughts and behaviors, substance use, and violence related behaviors and experiences among U.S. high school students
Source: PLOS Ment Health. 2025 Sep 17;2(9):e0000409. doi: 10.1371/journal.pmen.0000409 (PMC12724680; doi:10.1371/journal.pmen.0000409)
Supplement: S1 Fig — Test of parallel trend assumption - Trends in mental health, suicidal thoughts and behaviors, substance use, and violence related behaviors and experiences by school learning model in the 2020–2021 school year among Youth Risk Behavior Survey respondents in local jurisdictions with data from all survey years from 2013-2021. (PPTX) [file pmen.0000409.s003.pptx]

## Slide 1
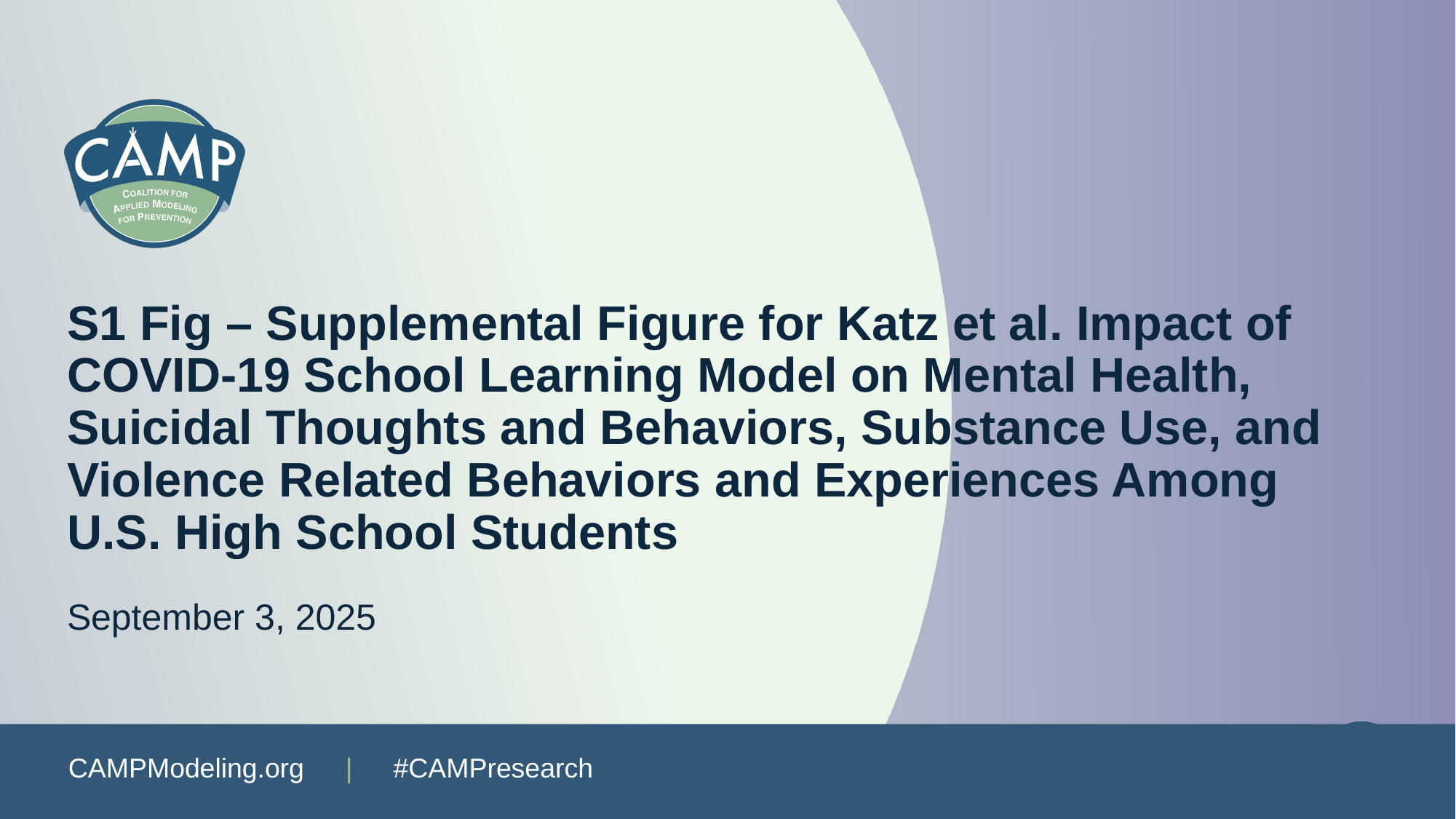

# S1 Fig – Supplemental Figure for Katz et al. Impact of COVID-19 School Learning Model on Mental Health, Suicidal Thoughts and Behaviors, Substance Use, and Violence Related Behaviors and Experiences Among U.S. High School Students
September 3, 2025

## Slide 2
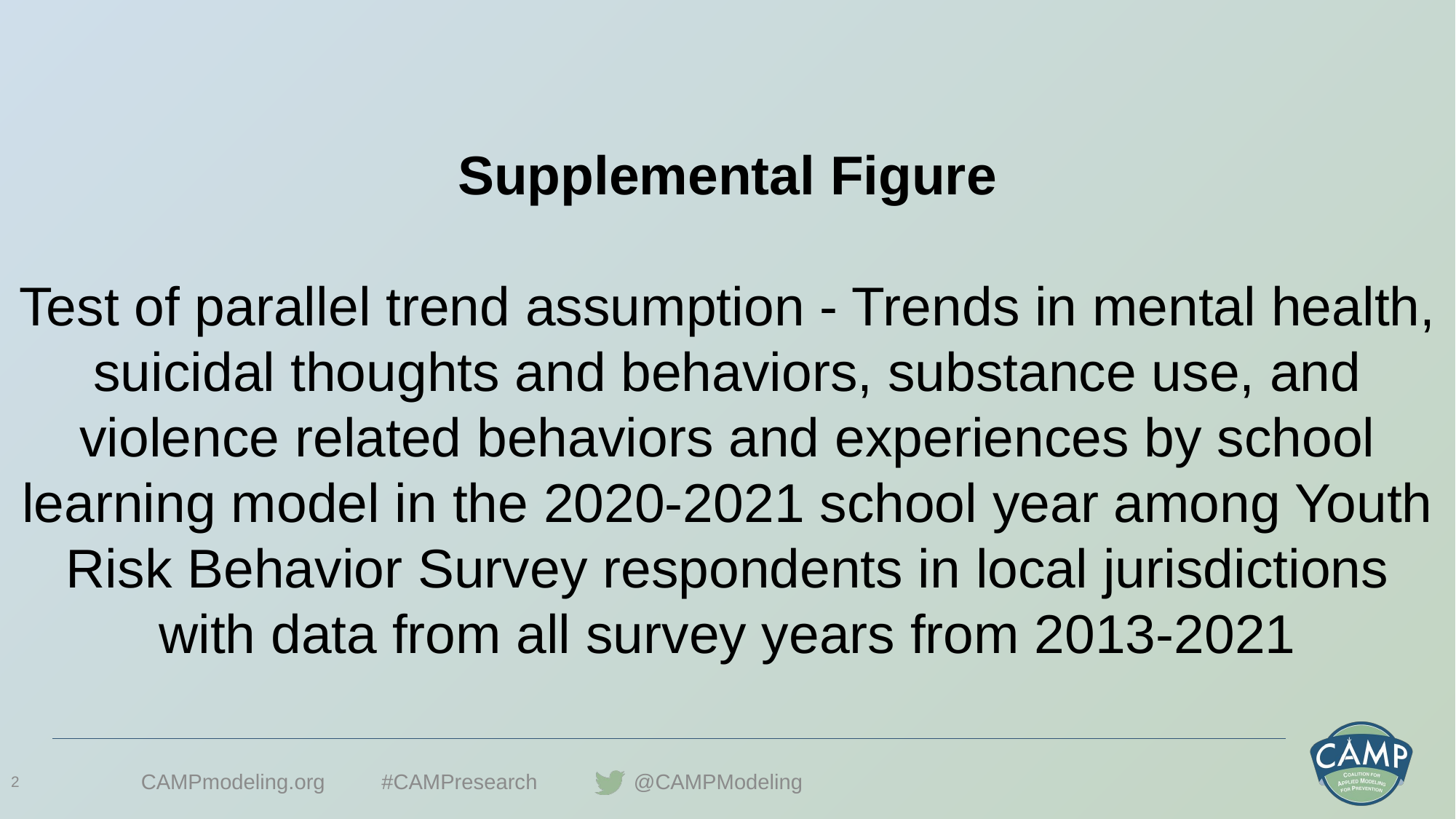

Supplemental Figure
Test of parallel trend assumption - Trends in mental health, suicidal thoughts and behaviors, substance use, and violence related behaviors and experiences by school learning model in the 2020-2021 school year among Youth Risk Behavior Survey respondents in local jurisdictions with data from all survey years from 2013-2021
2

## Slide 3
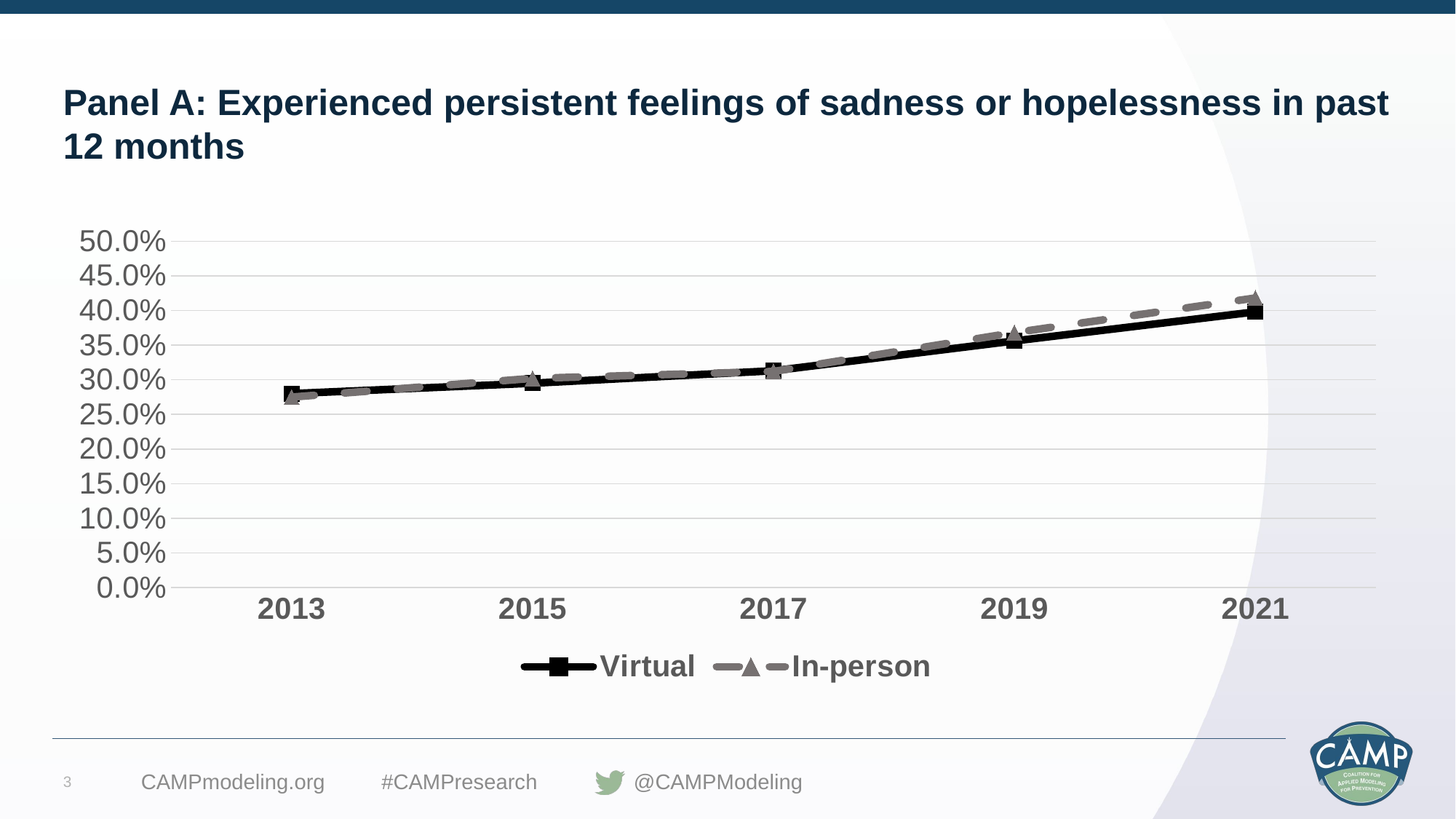

# Panel A: Experienced persistent feelings of sadness or hopelessness in past 12 months
### Chart
| Category | Virtual | In-person |
|---|---|---|
| 2013 | 0.28 | 0.275 |
| 2015 | 0.295 | 0.302 |
| 2017 | 0.313 | 0.312 |
| 2019 | 0.356 | 0.368 |
| 2021 | 0.398 | 0.418 |3

## Slide 4
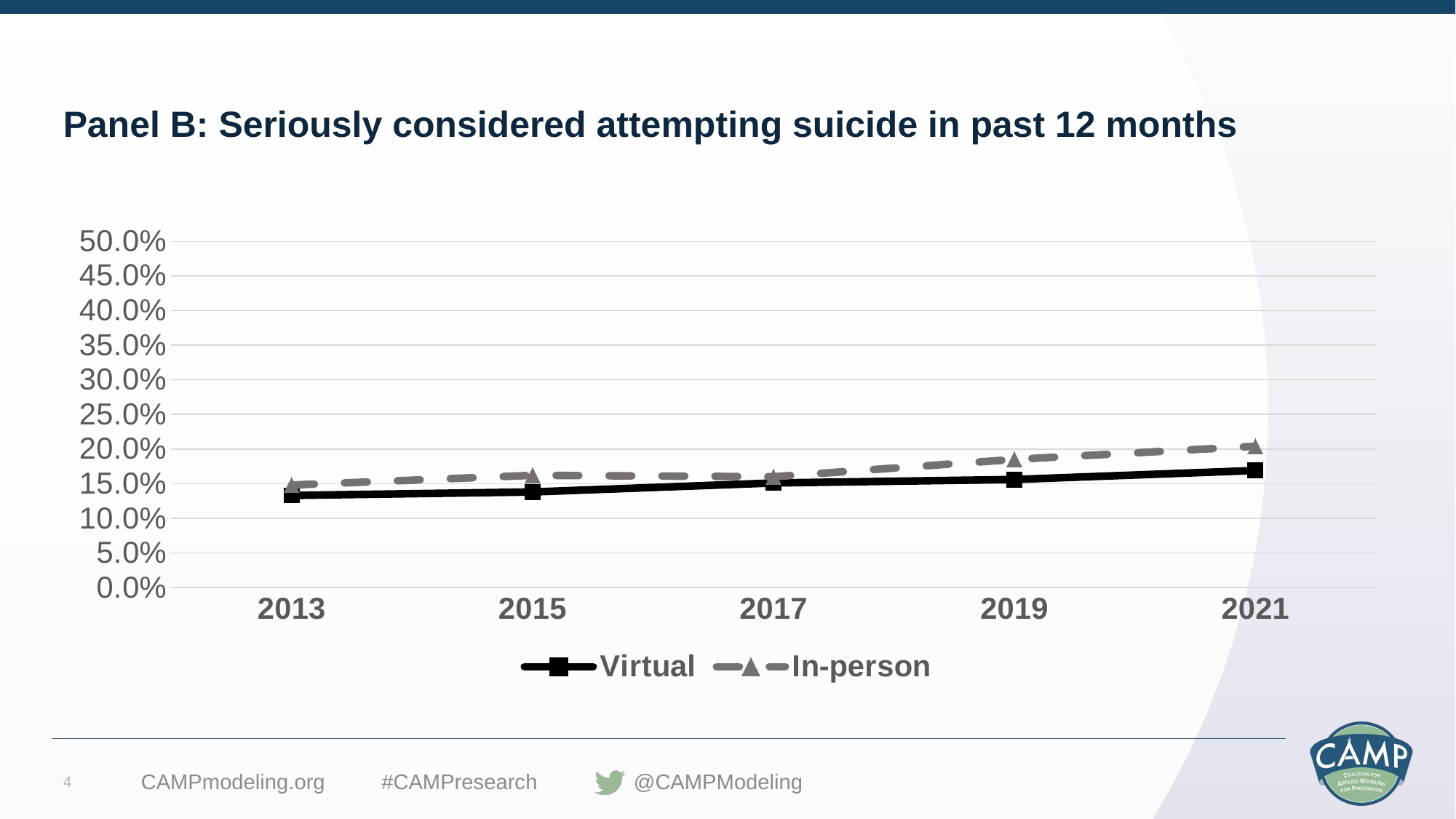

# Panel B: Seriously considered attempting suicide in past 12 months
### Chart
| Category | Virtual | In-person |
|---|---|---|
| 2013 | 0.133 | 0.148 |
| 2015 | 0.138 | 0.162 |
| 2017 | 0.151 | 0.16 |
| 2019 | 0.156 | 0.185 |
| 2021 | 0.169 | 0.204 |4

## Slide 5
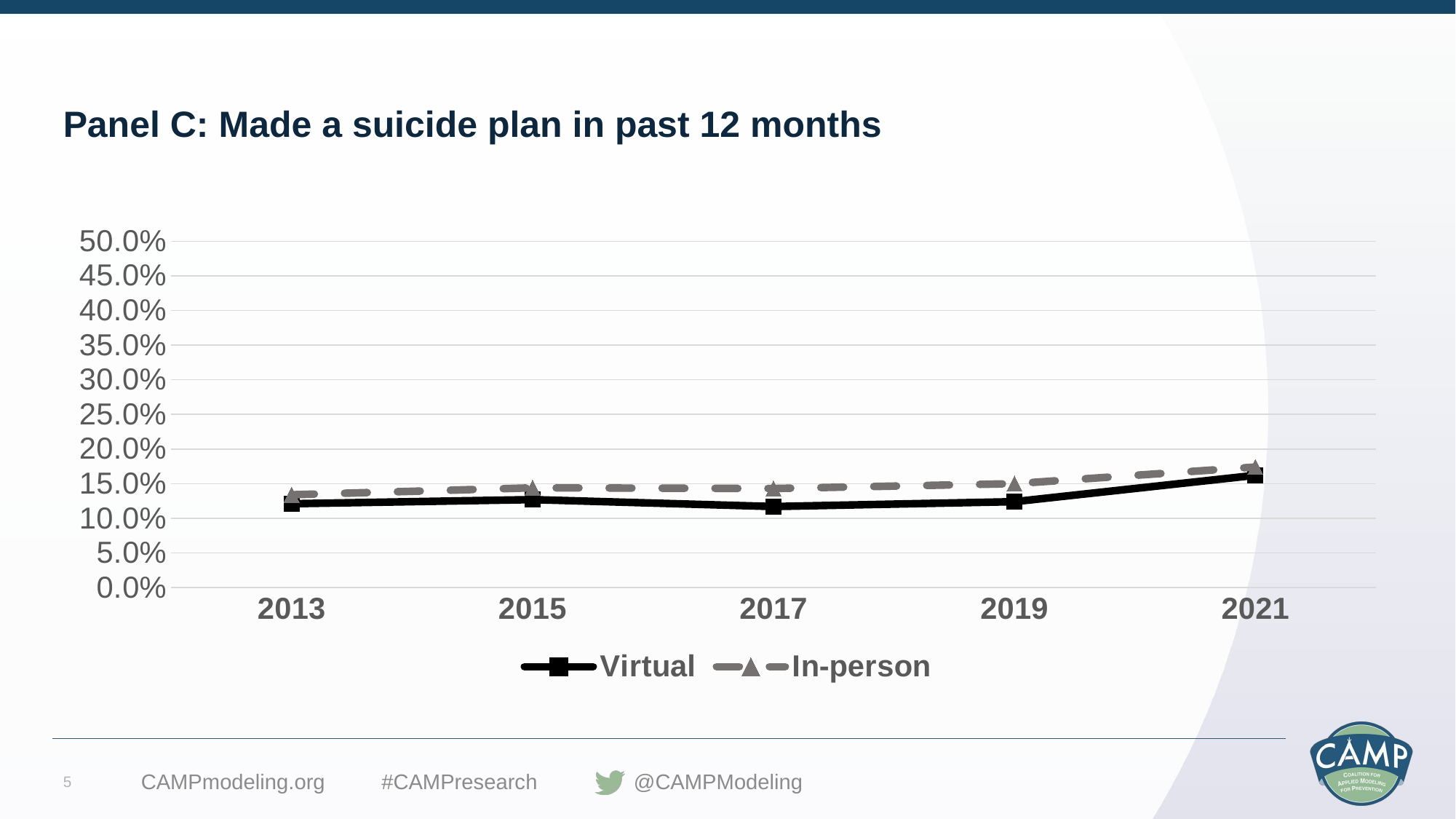

# Panel C: Made a suicide plan in past 12 months
### Chart
| Category | Virtual | In-person |
|---|---|---|
| 2013 | 0.121 | 0.134 |
| 2015 | 0.127 | 0.144 |
| 2017 | 0.117 | 0.143 |
| 2019 | 0.124 | 0.15 |
| 2021 | 0.162 | 0.174 |5

## Slide 6
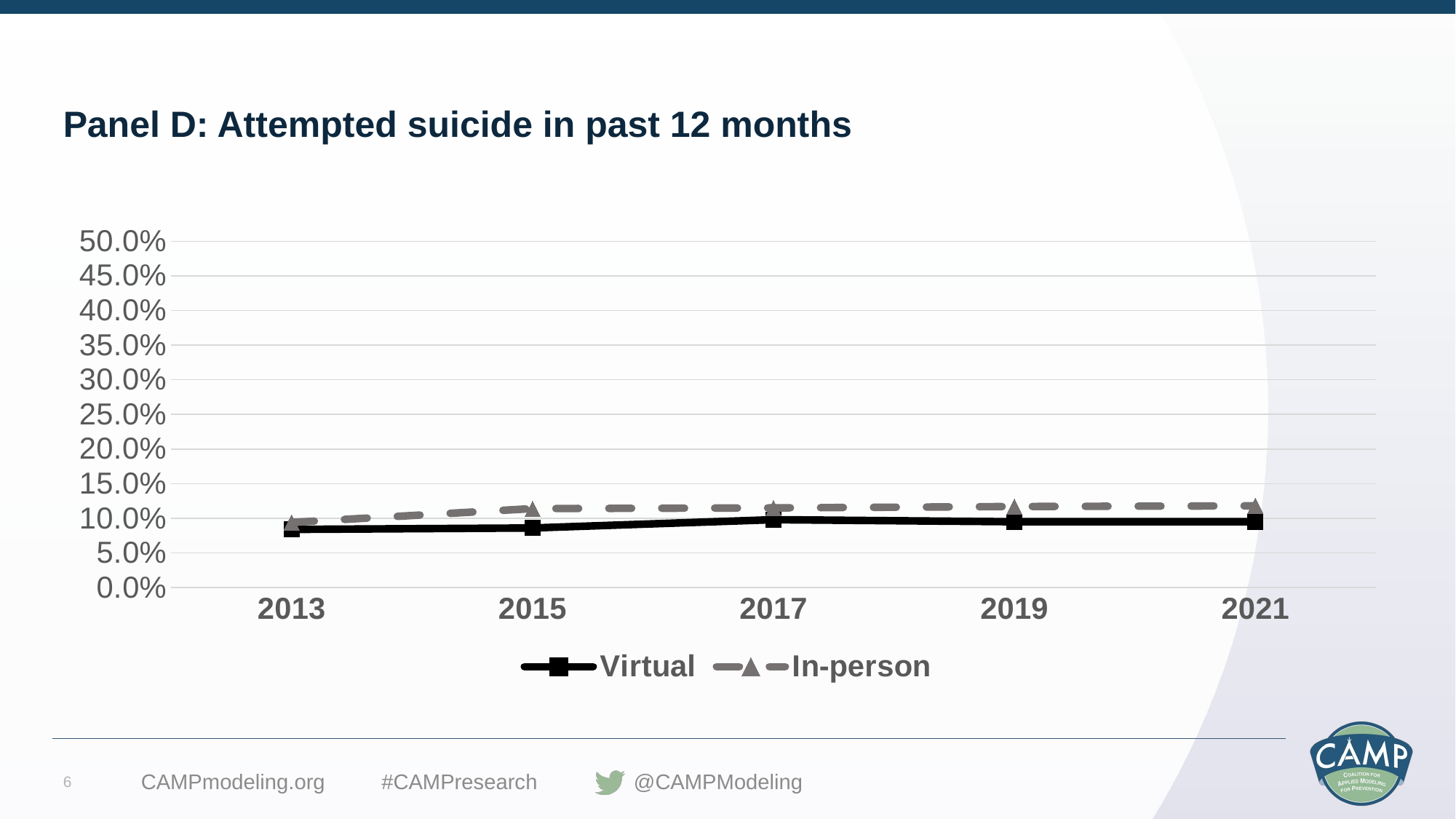

# Panel D: Attempted suicide in past 12 months
### Chart
| Category | Virtual | In-person |
|---|---|---|
| 2013 | 0.084 | 0.094 |
| 2015 | 0.086 | 0.114 |
| 2017 | 0.098 | 0.115 |
| 2019 | 0.095 | 0.117 |
| 2021 | 0.095 | 0.118 |6

## Slide 7
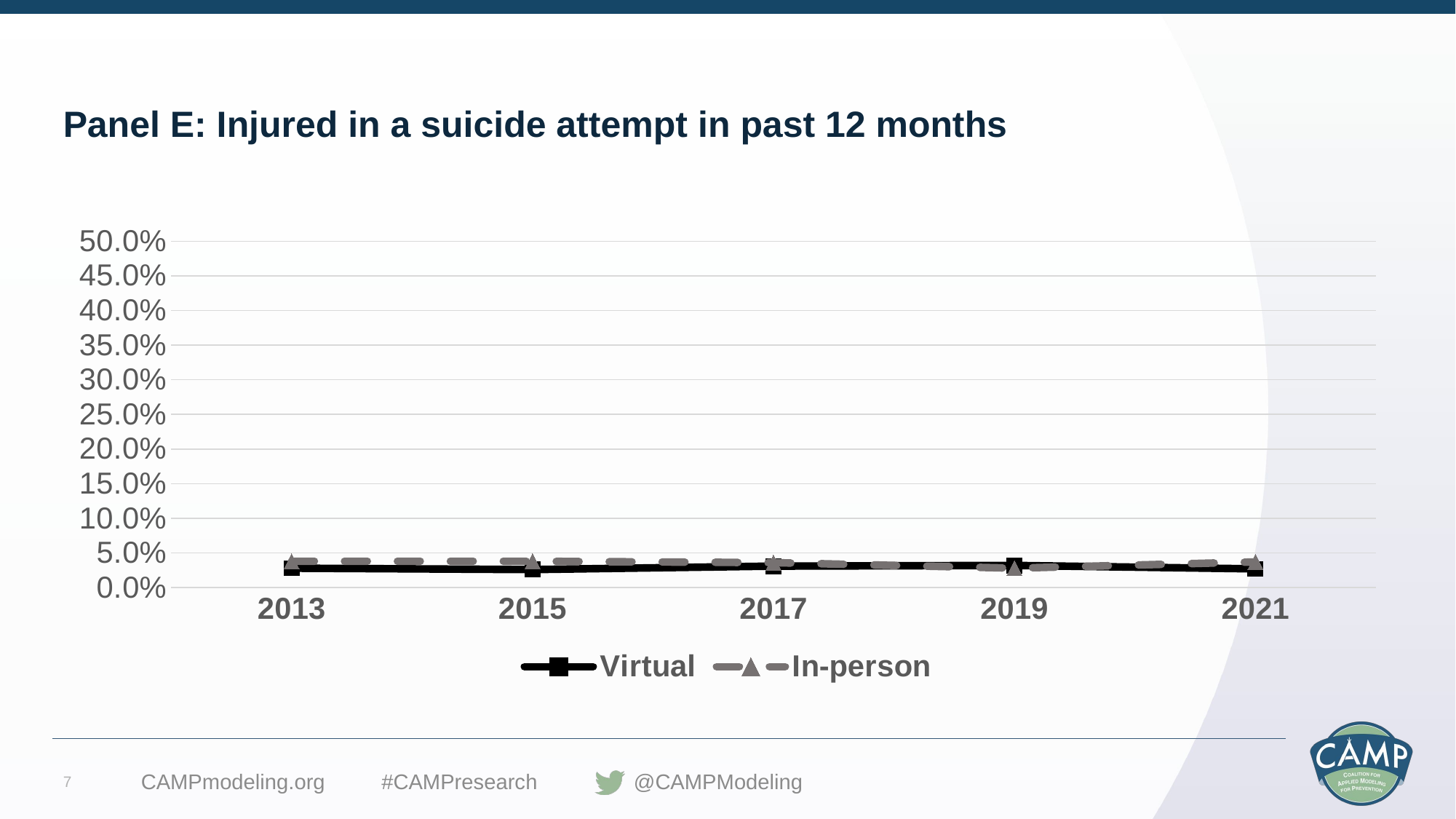

# Panel E: Injured in a suicide attempt in past 12 months
### Chart
| Category | Virtual | In-person |
|---|---|---|
| 2013 | 0.028 | 0.038 |
| 2015 | 0.026 | 0.038 |
| 2017 | 0.031 | 0.036 |
| 2019 | 0.032 | 0.028 |
| 2021 | 0.027 | 0.037 |7

## Slide 8
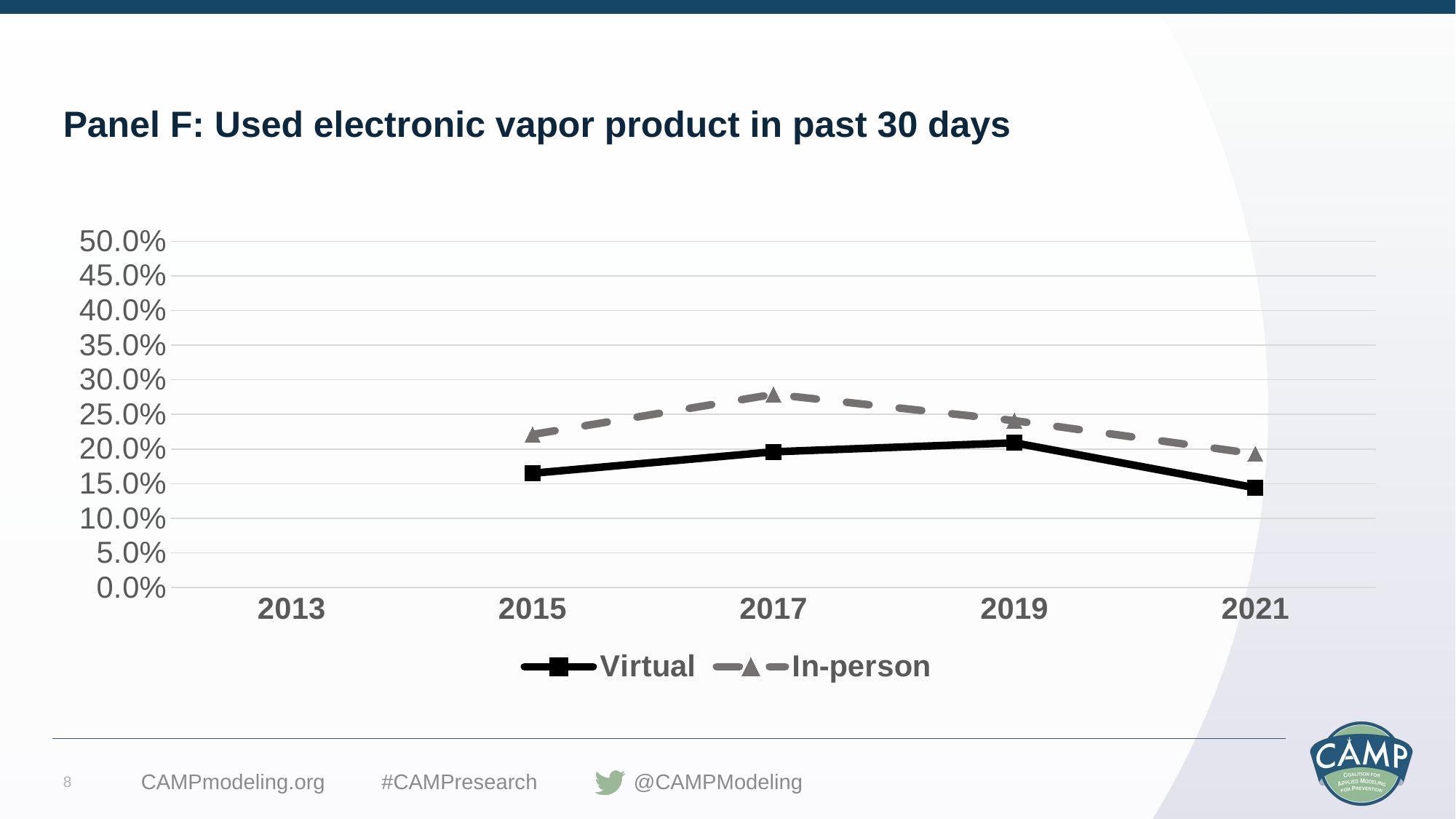

# Panel F: Used electronic vapor product in past 30 days
### Chart
| Category | Virtual | In-person |
|---|---|---|
| 2013 | None | None |
| 2015 | 0.165 | 0.221 |
| 2017 | 0.196 | 0.279 |
| 2019 | 0.209 | 0.241 |
| 2021 | 0.144 | 0.193 |8

## Slide 9
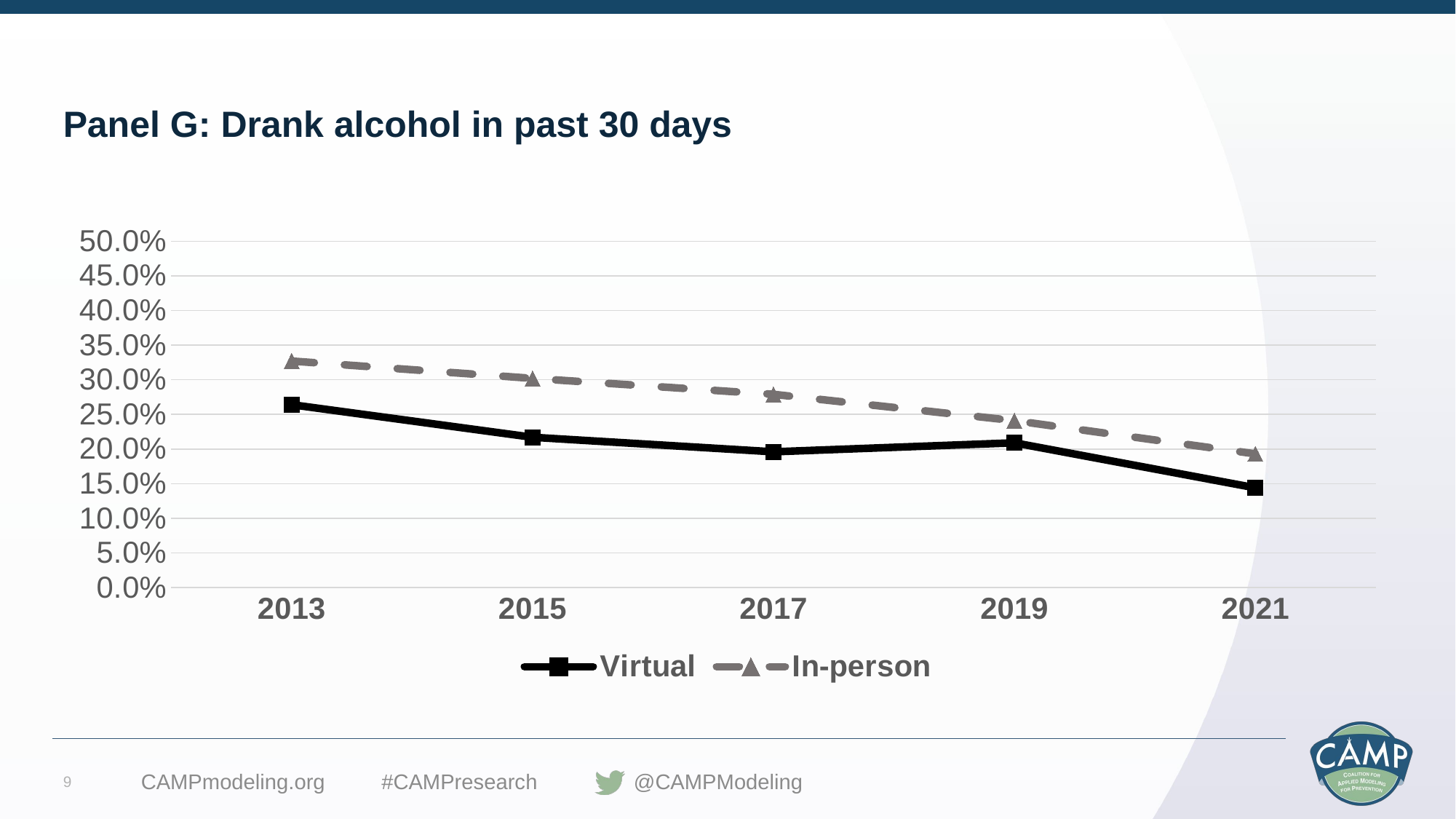

# Panel G: Drank alcohol in past 30 days
### Chart
| Category | Virtual | In-person |
|---|---|---|
| 2013 | 0.264 | 0.327 |
| 2015 | 0.217 | 0.302 |
| 2017 | 0.196 | 0.279 |
| 2019 | 0.209 | 0.241 |
| 2021 | 0.144 | 0.193 |9

## Slide 10
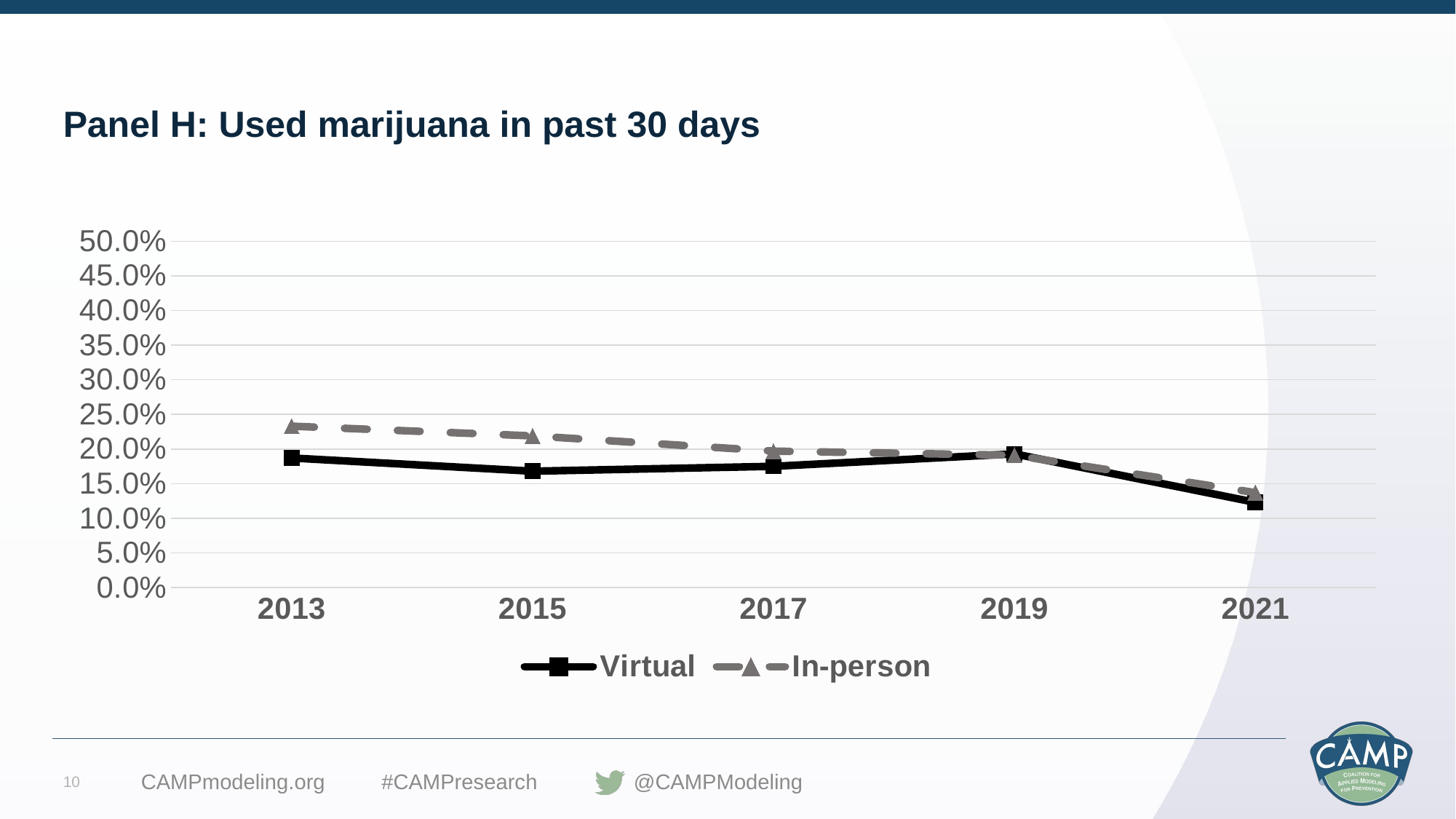

# Panel H: Used marijuana in past 30 days
### Chart
| Category | Virtual | In-person |
|---|---|---|
| 2013 | 0.187 | 0.233 |
| 2015 | 0.168 | 0.219 |
| 2017 | 0.175 | 0.197 |
| 2019 | 0.193 | 0.191 |
| 2021 | 0.123 | 0.137 |10

## Slide 11
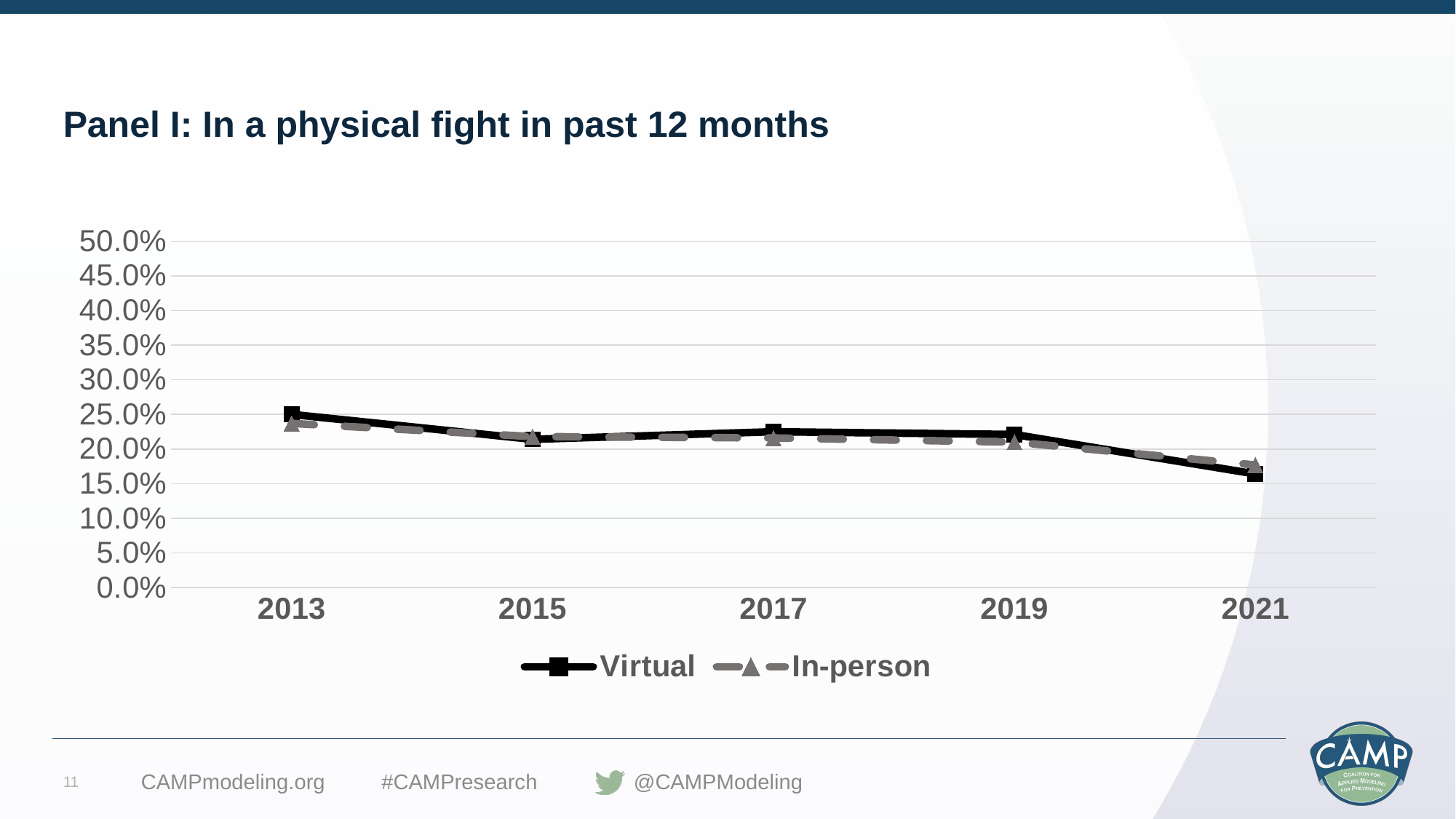

# Panel I: In a physical fight in past 12 months
### Chart
| Category | Virtual | In-person |
|---|---|---|
| 2013 | 0.25 | 0.237 |
| 2015 | 0.214 | 0.218 |
| 2017 | 0.225 | 0.216 |
| 2019 | 0.221 | 0.21 |
| 2021 | 0.164 | 0.177 |11

## Slide 12
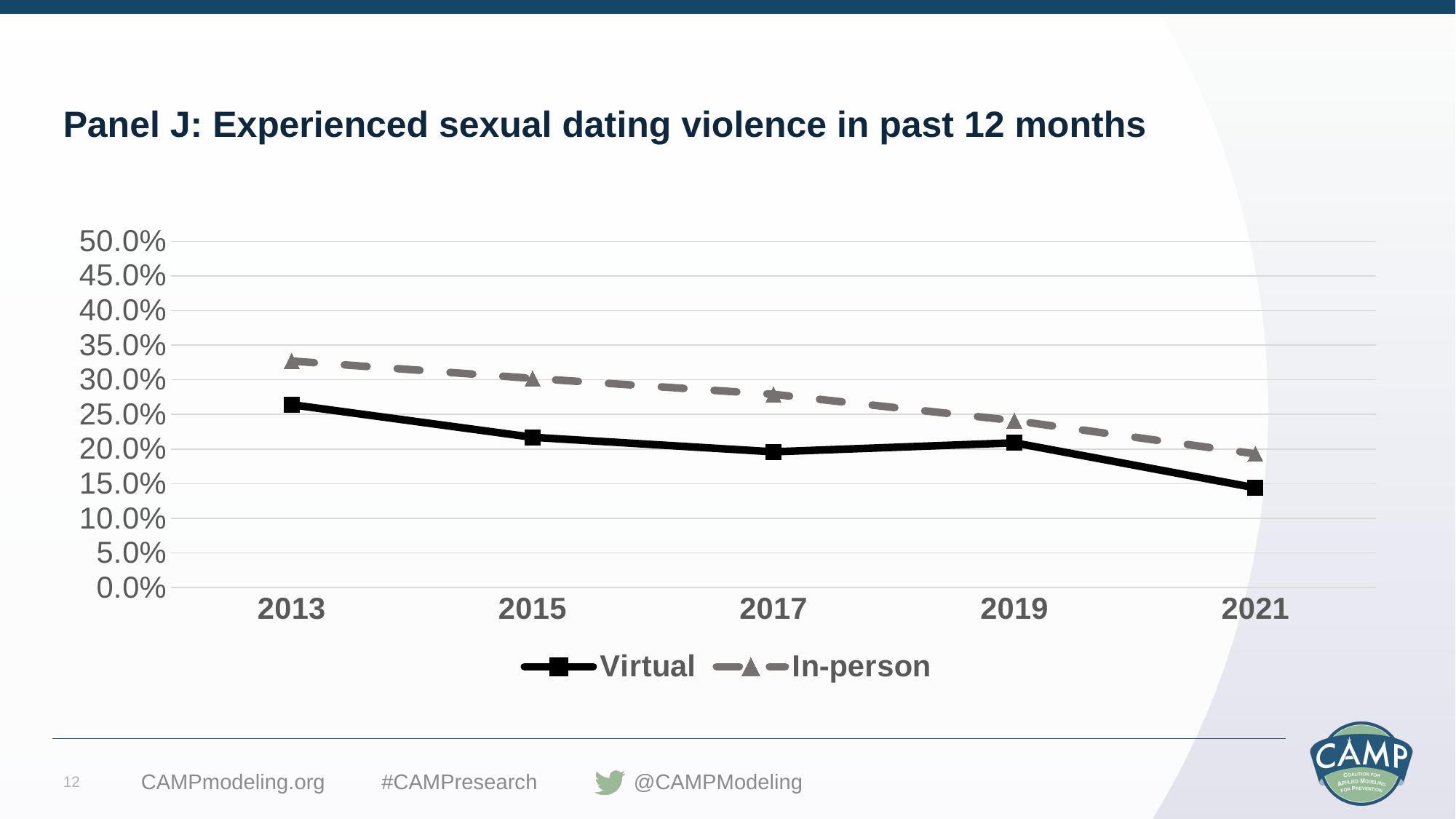

# Panel J: Experienced sexual dating violence in past 12 months
### Chart
| Category | Virtual | In-person |
|---|---|---|
| 2013 | 0.264 | 0.327 |
| 2015 | 0.217 | 0.302 |
| 2017 | 0.196 | 0.279 |
| 2019 | 0.209 | 0.241 |
| 2021 | 0.144 | 0.193 |12

## Slide 13
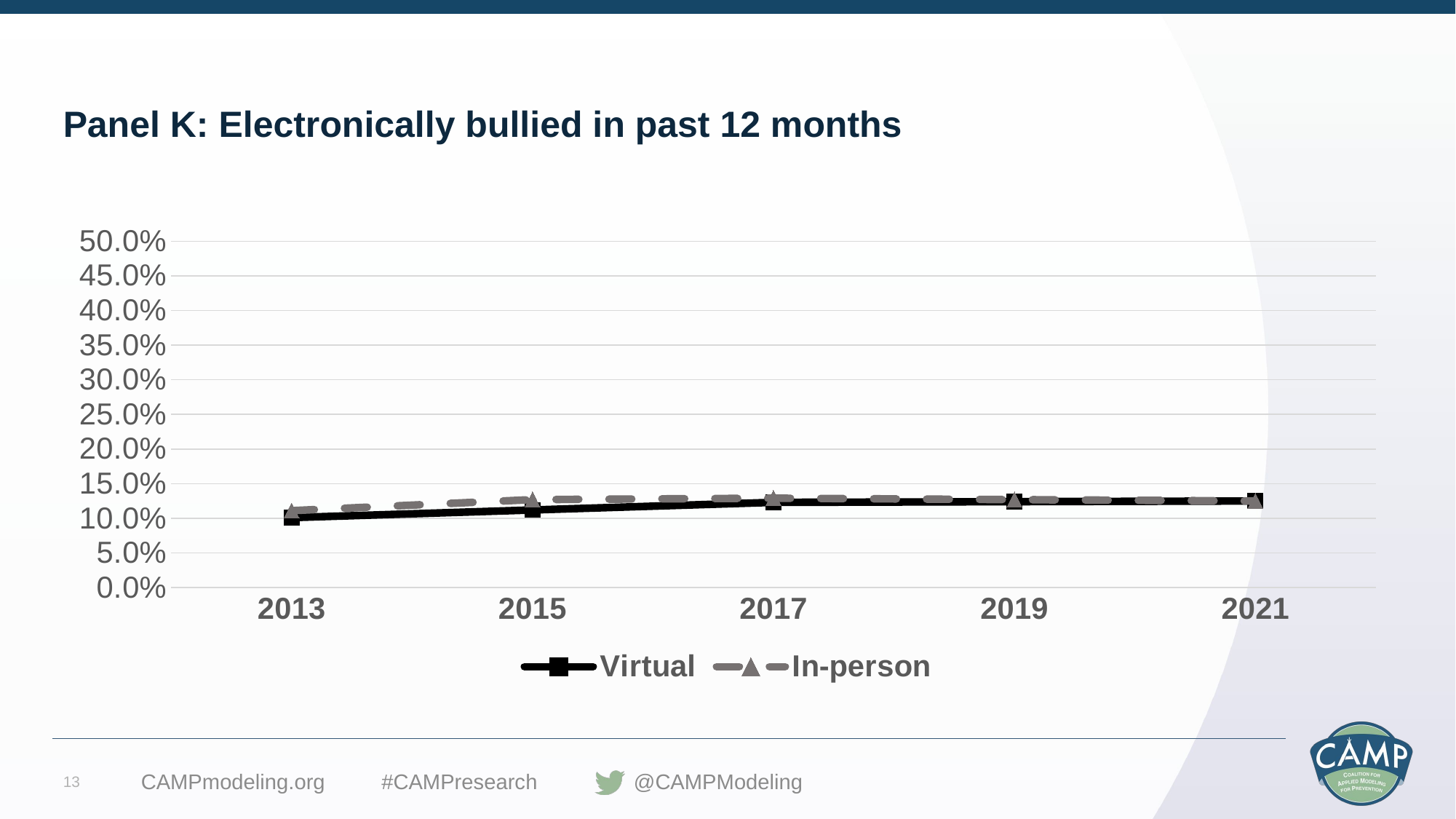

# Panel K: Electronically bullied in past 12 months
### Chart
| Category | Virtual | In-person |
|---|---|---|
| 2013 | 0.101 | 0.111 |
| 2015 | 0.112 | 0.127 |
| 2017 | 0.123 | 0.129 |
| 2019 | 0.124 | 0.127 |
| 2021 | 0.125 | 0.125 |13
